# Ivangustin, a sesquiterpene lactone from *Inula japonica* Thunb., attenuates neuroinflammation through covalent inhibition of ubiquitin-conjugating enzyme UBE2L3

**DOI:** 10.1186/s13020-026-01488-9

**Published:** 2026-09-02

**Authors:** Mengyuan Qiao, Hanyue Gan, Yuying Shi, Shan Yang, Ting Liu, Xiaofei Shen, Wenbin Wu

**Affiliations:** 1 Department of Geriatrics, Hospital of Chengdu University of Traditional Chinese Medicine, Chengdu University of Traditional Chinese Medicine, No. 39 Shierqiao Road, Jinniu District, Chengdu, 610075 China; 2 TCM Prevention and Treatment of Metabolic and Chronic Diseases Key Laboratory of Sichuan Province, Hospital of Chengdu University of Traditional Chinese Medicine, Chengdu University of Traditional Chinese Medicine, No. 39 Shierqiao Road, Jinniu District, Chengdu, 610075 China; 3 College of Pharmacy, Chengdu University of Traditional Chinese Medicine, Chengdu, 611137 China

**Keywords:** Ivangustin, Neuroinflammation, Microglia, UBE2L3, NEMO ubiquitination, NF-κB

## Abstract

**Background:**

Neuroinflammation is a key driver and an important therapeutic target of neurodegeneration. Dysregulated nuclear factor-κB (NF-κB) signaling plays a central role in neuroinflammation and depends on M1 and K63 ubiquitination of NF-κB essential modulator (NEMO). Ivangustin (IVA) is a sesquiterpene lactone isolated from *Inula japonica* Thunb., a traditional Chinese medicine has been used to treat inflammatory diseases. But its anti-neuroinflammatory effects and molecular targets remain unclear.

**Purpose:**

This study aimed to evaluate the anti-neuroinflammatory effects of IVA and elucidate its direct functional target and underlying mechanism.

**Methods:**

Integrated in vitro, in vivo, and in silico/bioinformatics approaches were employed. The anti-neuroinflammatory activity of IVA was evaluated in LPS-stimulated BV2 cells, primary microglia, and an LPS-induced acute cognitive impairment mouse model. Mechanistic studies were performed using pull-down, liquid chromatography-tandem mass spectrometry (LC–MS/MS), cellular thermal shift assay (CETSA), drug affinity responsive target stability (DARTS), gene knockdown, point mutation and bioinformatics analyses.

**Results:**

In vitro, IVA significantly inhibited nitric oxide (NO) production and mRNA and protein expression of pro-inflammatory factors in LPS-stimulated microglia. Furthermore, IVA significantly suppressed inhibitor of NF-κB kinase α/β (IKKα/β) and inhibitory subunit of NF-κB alpha (IκBα) phosphorylation, IκBα degradation, and NF-κB p65 phosphorylation and nuclear translocation, thereby blocking the expression of NF-κB-targeted genes. Its anti-neuroinflammatory activity was attenuated by thiol donors, indicating the importance of its electrophilic lactone moiety. Furthermore, ubiquitin conjugating enzyme E2L3 (UBE2L3) was identified as the functional target of IVA in BV2 cells, whereas UBE2L3 knockdown markedly attenuated IVA-mediated anti-neuroinflammatory action. Bioinformatics analysis suggests UBE2L3 overexpression as a potential risk factor for neurodegenerative diseases. Mechanistically, IVA covalently bound to the catalytic cysteine 86 of UBE2L3, thereby inhibiting UBE2L3-mediated M1 and K63 ubiquitination of NEMO, which is crucial for NF-κB activation. In vivo, IVA ameliorated LPS-induced cognitive impairment and neuroinflammation in mice via inhibiting Iba1 expression and suppressing NF-κB activation.

**Conclusion:**

IVA exerts anti-neuroinflammatory effects by covalently targeting UBE2L3 and suppressing NF-κB signaling. These findings also identify UBE2L3 as a potential therapeutic target for neuroinflammation and support IVA as a promising lead compound for further preclinical investigation of neuroinflammatory disorders.

**Graphical Abstract:**

**Figure:**
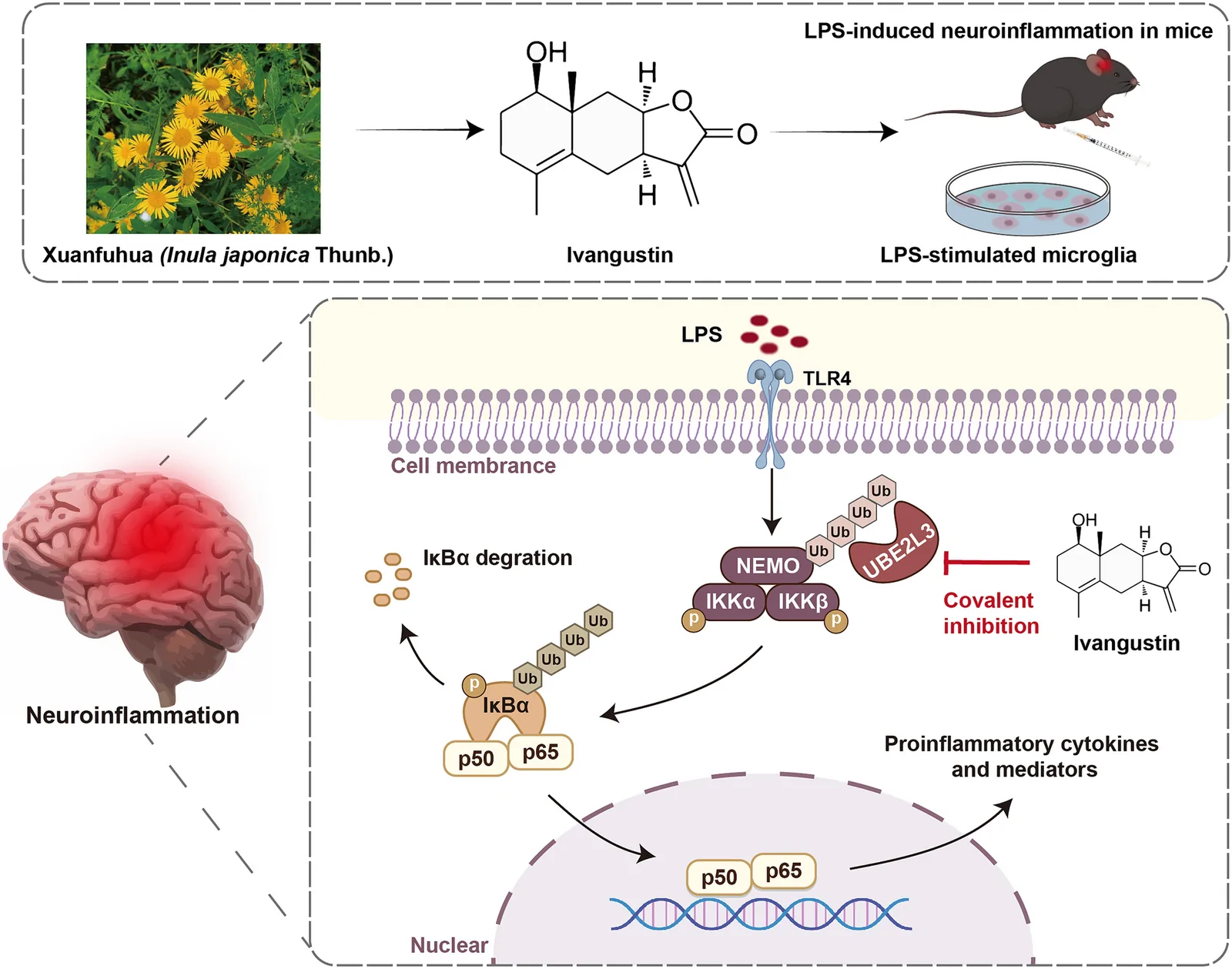

**Supplementary Information:**

The online version contains supplementary material available at https://doi.org/10.1186/s13020-026-01488-9.

## Introduction

Neurodegeneration refers to pathological conditions predominantly impacting neurons, characterized by progressive neuronal dysfunction and loss. Clinically, neurodegenerative diseases encompass a broad spectrum of central nervous system (CNS) disorders with distinct clinical and pathological features, including Alzheimer’s disease (AD), Parkinson’s disease (PD), amyotrophic lateral sclerosis (ALS), and Huntington’s disease (HD) [1, 2]. Despite disease-specific protein aggregates (e.g., amyloid-β, α-synuclein), all share a persistent neuroinflammation marked by elevated pro-inflammatory cytokines/chemokines, oxidative stress, and compromised neuroprotective mechanisms. This neuroinflammatory condition further exacerbates protein misfolding and directly induces synaptic damage and vascular impairment, creating a vicious cycle that accelerates disease progression [3, 4]. Thus, neuroinflammation serves as both a common pathological hallmark and a potential therapeutic target across neurodegenerative disorders [3–5].

As the CNS's resident immune cells, microglia maintain neural homeostasis under physiological conditions by continuously surveilling the microenvironment and clearing apoptotic cells and abnormal protein aggregates [6]. Pathologically, microglial activation is a key feature of neurodegenerative pathologies, where inflammatory stimuli trigger a substantial release of pro-inflammatory cytokines/chemokines, nitric oxide (NO), and reactive oxygen species (ROS) via activation of intracellular pro-inflammatory signaling pathways such as nuclear factor kappa B (NF-κB) and mitogen-activated protein kinases (MAPKs) [7, 8]. The centrality of this microglia-mediated neuroinflammation in the pathogenesis of neurodegenerative diseases has spurred therapeutic strategies targeting pro-inflammatory phenotype modulation, particularly NF-κB inhibition [9, 10]. Current approaches to inhibit NF-κB signaling primarily involve small molecules targeting upstream activators, such as BAY11-7082 and ainsliadimer A which directly inhibit inhibitor of NF-κB kinase β (IKKβ) kinase [11, 12]. Furthermore, NF-κB pathway activation also involves a cascade of ubiquitination events; notably, the formation and activation of the IKK complex requires polyubiquitination of the NF-κB essential modulator (NEMO) subunit, which recruits and facilitates phosphorylation of IKKα/β [13]. Consequently, modulating NEMO ubiquitination presents another potential strategy to inhibit IKKβ/NF-κB signaling cascades, offering a novel avenue for treating neurodegenerative diseases by suppressing neuroinflammation [13, 14].

Plant-derived active natural compounds remain a major source for new drug discovery [15, 16]. Sesquiterpene lactones, a class of plant secondary metabolites, exhibit potent bioactivities including anti-inflammatory, antioxidant, antitumor, and neuroprotective effects [17]. The most renowned example is artemisinin and its derivatives, which are marketed antimalarial and autoimmune disease therapeutics [18]. *Inula japonica* Thunb., rich in characteristic sesquiterpene lactones, is traditionally employed in traditional Chinese medicine (TCM) to treat diverse inflammatory conditions. Ivangustin (IVA), a sesquiterpene lactone isolated from the TCM herb Xuanfuhua (*Inula japonica* Thunb.), has been demonstrated anti-inflammatory potential by inhibiting lipopolysaccharide (LPS)-induced NO production in RAW264.7 macrophages [19]. It also exhibited moderate inhibitory effects against HCT116 colorectal and QGY7701 hepatocellular carcinoma cells [19]. However, its role in neuroinflammation and functional targets remains unknown. This study revealed that IVA significantly suppressed microglial activation and neuroinflammatory responses in vitro and in vivo. Mechanistically, IVA covalently modified the ubiquitin-conjugating enzyme E2L3 (UBE2L3), thereby inhibiting NEMO ubiquitination and subsequent IKKβ/NF-κB signaling cascade activation, ultimately exerting anti-neuroinflammatory effects. This discovery positions IVA as a promising neuroprotective agent, demonstrating the therapeutic potential of plant-derived ubiquitin modulators for inflammatory diseases.

## Materials and methods

### Reagents

**Fig. 1:**
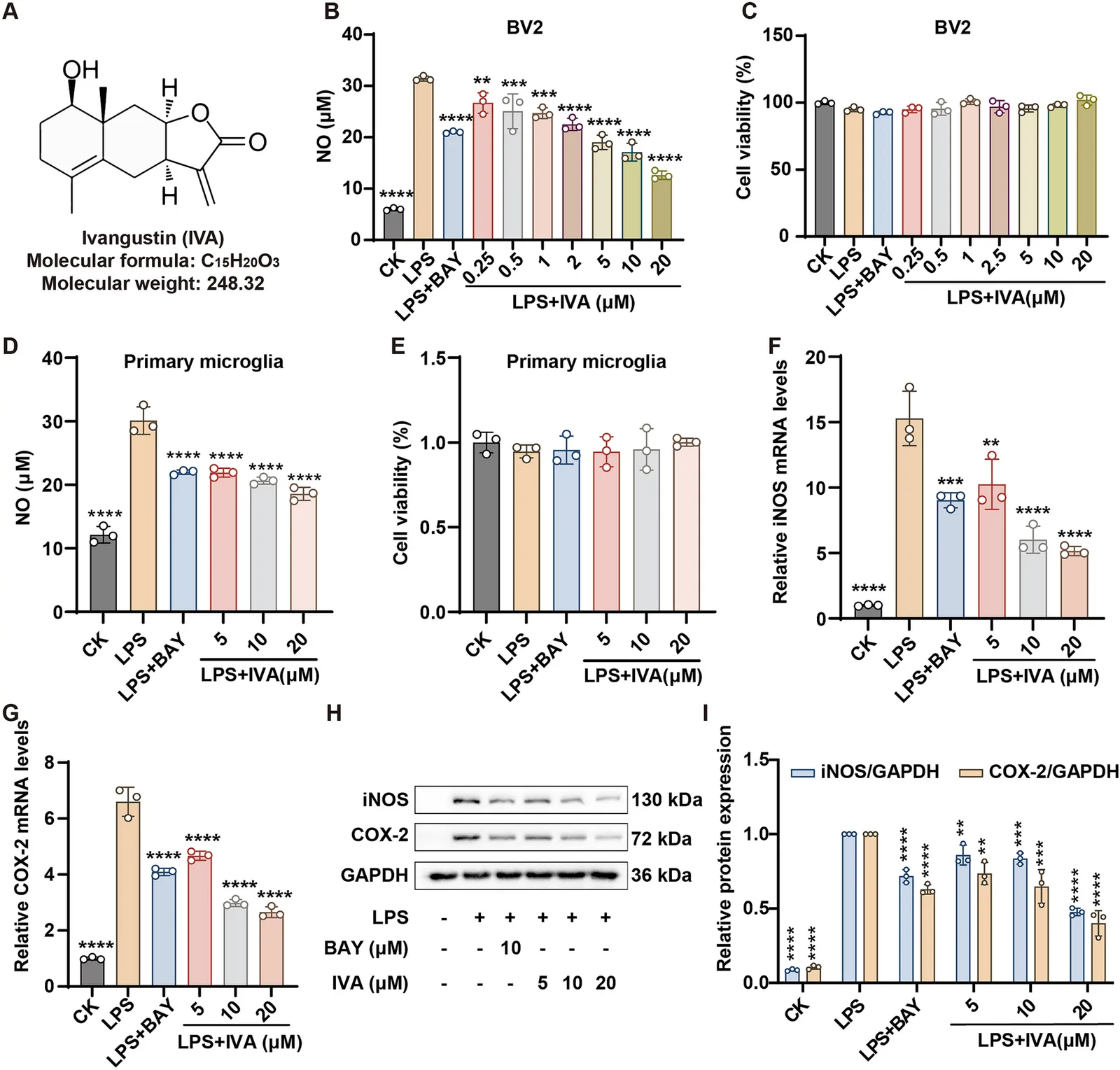
Anti-neuroinflammatory effects of IVA in LPS-stimulated microglia. **A** Chemical structure of ivangustin (IVA). **B** BV2 cells were pretreated with IVA or BAY (positive control) for 1 h, followed by stimulation with LPS (0.5 μg/mL) for 24 h, and NO production was quantified using the Griess reagent. **C** Cell viability of BV2 cells was then detected by the CCK8 reagent. **D** Primary microglia were pretreated with IVA for 1 h, followed by LPS (0.5 μg/mL) stimulation for 24 h, and NO production was measured using the Griess reagent. **E** Cell viability of primary microglia was detected by the CCK-8 reagent. **F**, **G** After IVA pretreatment for 1 h and subsequent LPS stimulation for 12 h, mRNA expression levels of iNOS (**F**) and COX-2 (**G**) were assessed via RT-qPCR. **H**, **I** IVA-pretreated BV2 cells were stimulated with LPS for 12 h, and protein levels of iNOS and COX-2 were analyzed by western blotting, with quantitative data showing iNOS/COX-2 expression normalized to GAPDH. *p < 0.05, **p < 0.01, ***p < 0.001, **** *p* < 0.0001, vs. LPS, n = 3

Ivangustin (IVA, Fig. 1A) was purchased from Sichuan ChemConst Biotechnology (Chengdu, China). HPLC analysis demonstrated the purity of IVA to be 98.087% (Supplementary Fig. 1). Dimethyl fumarate (DMF), SP600125 (SP, 20 μM, a selective JNK inhibitor), and SB203580 (SB, 20 μM, a selective p38 inhibitor) were obtained from TargetMol (Shanghai, China). Dimethyl fumarate (DMF, 100 mg/kg) was used as the in vivo positive control, based on its FDA-approved use for multiple sclerosis [20] and its reported mechanism of inhibiting UBE2L3-mediated NF-κB activation[21], making it mechanistically relevant to our study. BAY11-7082 (BAY, 10 μM, a NF-κB pathway inhibitor used as in vitro positive control) was provided by Beyotime (Shanghai, China). LPS isolated from *Escherichia coli* (O111:B4) was provided by Sigma-Aldrich (Shanghai, China). All concentrations were selected based on our preliminary experiments and previously reported studies [22, 23].

IVA, DMF, SB, SP, BAY was dissolved in dimethyl sulfoxide (DMSO) to prepare a 50 mM stock solution, thoroughly mixed by vortexing, and further homogenized by centrifugation. The stock solution was aliquoted (200 μL per tube) and stored at − 80 °C to minimize freeze–thaw cycles. Working concentrations were freshly diluted in culture medium prior to each experiment, with the final DMSO concentration maintained at 0.1% (v/v) in all in vitro assays, including the vehicle control (CK) group.

Antibodies (Ab) against inducible nitric oxide synthase (iNOS), cyclooxygenase 2 (COX-2), NEMO, GAPDH, and Tubulin were the products of ProteinTech (Wuhan, China). Antibodies targeting phosphorylated IKKα/β (p-IKKα/β), IKKα/β, p-inhibitory subunit of NF-κB alpha (p-IκBα), IκBα, p-p38, p38, p-c-Jun N-terminal kinase (p-JNK), and JNK were purchased from Beyotime (Shanghai, China). Antibody against Lysine 63 (K63)-linked ubiquitination (Ub) was supplied by Zen-Bio (Chengdu, China). Antibodies against Methionine 1 (M1)-linked ubiquitination and UBE2L3 were provided by ABclonal (Wuhan, China). Mouse IL-6 and TNF-α ELISA kits were purchased from Solarbio (SEKM-0007 and SEKM-0034 respectively; Beijing, China).

### Cell lines and cell culture

The BV2 mouse microglia cell line was obtained from Procell (Wuhan, China) and cultured in Dulbecco’s Modified Eagle’s Medium (DMEM, Gibco, USA) supplemented with 25 mM glucose, 10% fetal bovine serum (FBS, PAN Biotech, Germany), and 1% penicillin/streptomycin solution (Gibco, USA). Cells were maintained in a humidified incubator at 37 °C with 5% CO₂.

Primary microglia were isolated from the cerebral cortices of postnatal day 1 C57BL/6 mouse pups as previously described [24]. Briefly, cortices were dissected, minced, and digested with 0.25% trypsin–EDTA for 15 min at 37 °C. The dissociated cells were filtered through a 70 μm cell strainer and cultured in the same DMEM medium supplemented with 10% FBS and 1% penicillin/streptomycin. After 12 days in culture, microglia were harvested by gently tapping and purified by differential adhesion.

### Nitric oxide (NO) detection and cell viability assay

Briefly, BV2 cells were seeded into 96-well plates at a density of 7,500 cells per well and allowed to adhere overnight. Subsequently, cells were treated with IVA at varying concentrations in the presence of LPS (0.5 μg/mL) for 24 h. The cell culture supernatant was then collected by centrifugation at 1,000×*g* for 3 min at 4 °C. The supernatant was then subjected to NO quantification using the Griess reagent (Beyotime, Shanghai, China).

For cell viability assessment, BV2 cells were then incubated with 100 μL of fresh medium containing 1% CCK-8 reagent (Beyotime) at 37 °C protected from light for 1 h. Subsequently, the optical density (OD) of each well was measured at 450 nm using a multifunctional microplate reader (Tecan, Innsbruck, Austria).

### Quantitative real time polymerase chain reaction (qRT-PCR)

Following LPS stimulation for 12 h, total RNA was extracted from BV2 cells using the Steady Pure RNA extraction kit (Accurate, Changsha, China). cDNA synthesis was performed using the Evo M-MLV Reverse Transcription Kit (Accurate) to convert extracted RNAs into cDNAs. qRT-PCR was subsequently conducted on a LineGene 9600 Plus system (Bioer Technology, Hangzhou, China) with SYBR Green Pro Taq HS qPCR Mix (Accurate). Custom-designed primers (Tsingke, Beijing, China) were employed for amplification. Target gene expression levels were normalized to GAPDH as the internal reference and quantitatively analyzed using the 2^−ΔΔ^Ct method. The sequences of PCR primers used in this study are listed in Supplementary Table 1.

### Western blotting

Following DMSO or IVA treatment, BV2 cells were lysed in RIPA buffer containing 1% protease/phosphatase inhibitors (APExBio) for 15 min on ice. After centrifugation at 4 °C (15,000*g*, 5 min), supernatants were collected and boiled with 5 × loading buffer (10 min, 100 °C). Proteins were then resolved by SDS-PAGE and transferred to ethanol-activated polyvinylidene fluoride (PVDF) membranes (Millipore, USA). Membranes were blocked with 5% skim milk in Tris-buffered saline with Tween 20 (TBST) for 1 h at 25 °C, then probed with primary antibodies (1:1000 diluted in 5% bovine serum albumin) overnight at 4 °C. After TBST washes, horseradish peroxidase (HRP)-conjugated secondary antibody (1:5000, SAB, Nanjing, China) was applied to bind the primary antibodies for 1 h at 25 °C. Signals were detected using chemiluminescence reagent (Oriscience Biotechnology) on a MiniChemi™ 610 system (Sage, Beijing), with band intensities quantified by ImageJ (NIH, USA).

### Subcellular localization analysis of p65

DMSO- or IVA-treated BV2 cells were stimulated with LPS for 30 min. Cytoplasmic and nuclear fractions were isolated using a commercial kit (Beyotime). Western blotting was performed to assess the abundance of p65 in each fraction, with PARP1 (nuclear) and GAPDH (cytoplasmic) as loading controls.

### Covalent conjugation of IVA with β-mercaptoethanol (BME)

In brief, IVA (20 μM) was incubated with BME (100 μM) at 37 °C for 60 min, and the resulting mixture was analyzed by liquid chromatography-mass spectrometry (LC–MS) system (Waters Vion® IMS Q-tof, Manchester, UK) in ESI-positive mode with the following parameters: mobile phase (methanol/water, 1:1, 0.2 mL/min), capillary voltage (3.0 kV), source temperature (120 °C), desolvation temperature (450 °C), cone gas flow (50 L/h), and desolvation gas flow (800 L/h).

### Pull-down assay

Based on the approach reported by Chen et al. [25, 26], an improved method is applied for target identification. BV2 cells were lysed on ice using immunoprecipitation (IP) buffer (Beyotime) containing 1% protease inhibitor (APExBIO, Houston, USA) with sonication (50% power, 5 s on/5 s off intervals) for 3 min. The lysate was centrifuged at 12,000*g* for 5 min at 4 °C, and the supernatant was collected and quantified to a protein concentration of 1 μg/μL. The samples were equally divided into two groups and treated with IVA (200 μM) or an equal volume of DMSO at 4 °C for 6 h, followed by incubation with biotin-labeled iodoacetamide (Biotin-IA, 20 μM; MCE, Shanghai, China) for 1 h at 4 °C. Subsequently, 50 μL of streptavidin-coupled magnetic beads (Beyotime) were added to each group and incubated overnight at 4 °C. The beads were then collected and washed six times with IP buffer containing 1% protease inhibitor (5,000*g*, 1 min per wash at 4 °C). The samples were used for MS detection or western blotting analysis.

### LC–MS/MS assay

For protein digestion, the bead-bound proteins were incubated with 5 mM dithiothreitol at 56 °C for 30 min and alkylated with 11 mM IA at 25 °C for 15 min in darkness. The protein was then diluted with 100 mM triethanolamine borate to urea concentration < 2 M before digestion with trypsin at 1:50 (w/w) overnight followed by 1:100 (w/w) for 4 h. Peptides were desalted by C18 SPE, dissolved in solvent A (0.1% formic acid, 2% acetonitrile in water), and analyzed on a homemade RP column (25 cm × 100 μm) using a NanoElute UHPLC system (Bruker Daltonics, Germany) with solvent B (0.1% formic acid in acetonitrile). Separation was performed with a gradient of 6–24% B (0–14 min), 24–35% B (14–16 min), 35–80% B (16–18 min), and 80% B (18–20 min) at 500 nL/min. The peptide samples were subjected to analysis using a timsTOF Pro 2 mass spectrometer (Bruker Daltonics, Germany) equipped with a 1.75 kV capillary source. The instrument operated in data-independent parallel accumulation serial fragmentation (dia-PASEF) mode, acquiring full MS scans over the mass range of 300–1500 m/z and MS/MS scans from 400–850 m/z. Each cycle included 20 PASEF scans with a 7 m/z isolation window.

The binding site of IVA on UBE2L3 was characterized through LC–MS/MS analysis. Recombinant human UBE2L3 (MCE, Shanghai, China) was incubated with 20 μM IVA or DMSO at 4 °C overnight. Following SDS-PAGE separation and Coomassie blue staining, UBE2L3-containing bands were excised and subjected to tryptic digestion. The resulting peptides were separated on an EASY-nLC 1200 system (Thermo Fisher Scientific) using a linear gradient of solvent A (0.1% formic acid, 2% acetonitrile) and solvent B (0.1% formic acid, 90% acetonitrile). Mass spectrometric analysis was performed on a Q Exploris 480 instrument (Thermo Scientific, USA) equipped with a nanospray ionization source (2.1 kV), acquiring data at 60,000 resolution in Orbitrap mode. MS data were processed using Proteome Discoverer 2.4 software for comprehensive analysis.

### Cellular thermal shift assay (CETSA)

BV2 cells were suspended in PBS containing 1% protease inhibitors and lysed using five freeze–thaw cycles in liquid nitrogen. After centrifugation (12,000*g*, 10 min, 4 °C), the supernatants were collected and quantified to a protein concentration of 1 μg/μL. The supernatants were then equally partitioned and incubated with either 100 μM IVA or DMSO control at 25 °C for 60 min. Each treated sample was then divided into seven aliquots and subjected to thermal challenge at 65–93 °C for 3 min. Following heat treatment, samples were centrifuged (12,000*g*, 10 min, 4 °C), supernatants boiled with loading buffer, and target protein levels quantified by western blotting analysis.

### Drug affinity response target stability (DARTS) assay

BV2 cells were lysed using M-PER buffer (Thermo Fisher Scientific, Waltham, USA) supplemented with 1% protease inhibitor. After centrifugation at 12,000*g* for 10 min at 4 °C, the supernatant was diluted with 10 × TNC buffer and quantified to a protein concentration of 1 μg/μL. The supernatants were then equally divided into two portions for incubation with either DMSO or 100 μM IVA, incubated at 25 °C for 60 min. The protein samples were subsequently digested with 0.4% pronase (Roche, Basel, Switzerland) for 30 min at 25 °C prior to western blotting analysis of the DARTS reaction products.

### UBE2L3 knockdown and point mutation experiments

The UBE2L3 siRNA and negative control (NC) sequences (Tsingke Biotech, Beijing, China) were transfected into BV2 cells using Lipo6000™ transfection reagent (Beyotime, Shanghai, China) in FBS-free DMEM medium for 48 h. Western blotting analysis was performed to evaluate the knockdown efficiency of UBE2L3 siRNA. Subsequently, the transfected BV2 cells were treated with IVA in the presence of LPS, NO release and NF-κB activation were then measured to assess the impact of UBE2L3 knockdown on IVA-mediated anti-neuroinflammatory effect. The sequences of UBE2L3 siRNA are described in Supplementary Table 2.

For point mutation, wild-type UBE2L3 (WT-UBE2L3) and C86S mutant UBE2L3 (C86S-UBE2L3) plasmids were constructed using the pcDNA3.1 vector and transfected into HEK293T cells (for pull-down binding assay) or transfected into UBE2L3-knockdown BV2 cells (for NO release assay) using Lipo6000™ transfection reagent for 24 h. The WT plasmid and mutant plasmids were constructed by HITRO BioTech (Tianjin, China).

### Co-immunoprecipitation (Co-IP) assay

LPS-stimulated BV2 cells were lysed in ice-cold IP buffer supplemented with 1% protease inhibitor. Following centrifugation at 12,000*g* for 10 min at 4 °C, the supernatants were subjected to immunoprecipitation using anti-NEMO antibody conjugated to protein A/G magnetic beads (Bimake, Shanghai, China) overnight at 4 °C. After three washes with IP buffer, the immunoprecipitated complexes were boiled and analyzed by western blotting with anti-Ub antibody to assess ubiquitination of NEMO.

### Molecular docking

The crystal structure of human UBE2L3 (AF-P68036-F1) and the chemical structure of IVA were retrieved from the AlphaFold2 and PubChem databases, respectively. Covalent docking was then employed to model the Michael addition reaction between the α-methylene-γ-lactone moiety of IVA and Cys86 of UBE2L3 using Autodock4.0 (Scripps, USA). Covalent docking was performed following AutoDock's recommended flexible residue covalent docking strategy. Briefly, the ligand and the reactive residue (Cys86) were connected via a covalent bond to construct the covalent adduct model, which was then set as flexible while the rest of the receptor remained rigid. AutoDockTools was used for hydrogen addition, charge assignment, and PDBQT format conversion, with the docking grid centered on the binding pocket (grid center: x = − 0.5768, y = − 0.2598, z = − 0.023). During docking, the covalent bond was fixed, and only the rotatable bonds within the covalent adduct and flexible residue side chains were sampled. Final molecular interactions were visualized using PyMOL (Schrödinger, New York, USA).

### Molecular dynamic (MD) simulation

The protein–ligand complex was subjected to 100 ns molecular dynamics simulations using GROMACS 2023. The CHARMM36 force field was employed for protein modeling, while the GAFF2 force field parameters were used to generate ligand topologies. The complex was placed in a cubic box with periodic boundary conditions and solvated using the TIP3P water model. Particle Mesh Ewald (PME) and Verlet algorithms were applied to handle electrostatic interactions. The system underwent 100,000 steps of equilibration in both NVT (constant particle number, volume, and temperature) and NPT (constant particle number, pressure, and temperature) ensembles, with a coupling constant of 0.1 ps for 100 ps. Van der Waals and Coulomb interactions were calculated using a cutoff distance of 1.0 nm. Finally, MD simulations were performed at a constant temperature of 300 K and 1 bar pressure for a total duration of 100 ns using GROMACS 2023.

### Bioinformatics analysis

Mendelian randomization (MR) was used to investigate the causal relationship between UBE2L3 gene expression and neurodegenerative diseases, using peripheral blood expression data of UBE2L3 from the eQTLGen Consortium as the exposure, and AD (ukb-r2-20110_10), PD (ukb-saige-332), and cognitive impairment (ukb-r2-R41) data from the UK Biobank as outcomes, respectively. The study identified eQTLs significantly associated with UBE2L3 expression (p < 5 × 10⁻⁸) as instrumental variables. Linkage disequilibrium (LD) clustering was performed using the 1000 Genomes European reference panel (r^2^ < 0.001, 10,000 kb window). For PD and cognitive impairment, a slightly relaxed LD threshold (r^2^ < 0.1) was applied to maintain statistical power, with all single nucleotide polymorphisms (SNPs) exhibiting F-statistics > 10 to mitigate weak instrument bias. Five Mendelian randomization methods were employed for causal estimation: inverse variance weighting (IVW) random-effects model as the primary approach, supplemented by MR-Egger, weighted median, simple mode, and weighted mode. Result robustness was assessed through Cochran's Q test, MR-Egger intercept test, and leave-one-out analysis. All analyses were conducted in R 4.3.1 using the TwoSampleMR package (v0.5.7).

Furthermore, microarray datasets GSE15222, GSE118553, and GSE7621 were downloaded from the Gene Expression Omnibus (GEO) database, covering normal brain tissues and samples from AD and PD. The raw expression data underwent preprocessing in R, including normalization and deduplication. Based on the preprocessed expression matrix, differential gene expression analysis and gene–gene correlation analysis were performed, with a focus on UBE2L3 as the target gene. The results were visualized to enhance interpretability and reveal potential biological relationships. Differential expression analysis was conducted using t-tests, while gene–gene correlations were assessed using Pearson correlation coefficients.

### Animals

Ten-week-old male C57BL/6 J mice (SPF grade, 26 ± 2 g) were purchased from Huafukang Biotechnology Co., Ltd (Beijing, China, SCXK-JING-2024-0003). Mice were housed in plastic cages at 25 ± 1 °C under a 12-h light/dark cycle for a 1-week acclimation period, with free access to food and water.

### Neuroinflammation induction and pharmacological intervention in mice

The mice were randomly assigned to six groups based on body weight: control group (vehicle), LPS group (vehicle), LPS + dimethyl fumarate group (DMF, 100 mg/kg), and LPS + three dose groups of IVA (5, 10, and 20 mg/kg). All groups received daily intraperitoneal injections of respective treatments (vehicle: 5% DMSO + 30% PEG400 + 65% saline) for four consecutive days. The doses of IVA were selected based on our laboratory's previous experience with structurally similar sesquiterpene lactones in vivo; IVA and DMF was completely soluble in the vehicle at all tested doses, ensuring accurate and stable administration. On day 4, LPS (2.5 mg/kg) was intraperitoneally administered to all groups except the vehicle control. Furthermore, body weight was monitored throughout the experimental period.

### Behavioral testing

Prior to behavioral assessment, mice were acclimated to the environment for 24 h. Testing was conducted under quiet conditions with appropriate lighting and experimenters remaining out of sight. Behavioral assessments were performed 16 h after LPS injection. For open-field test (OFT), mice were placed in the center of a 50 × 50 cm arena, and their movement trajectories, total distance traveled, average speed, and entries/distance in the central zone were recorded over 5 min to evaluate locomotor activity and exploratory behavior. For the Y-maze test, mice freely explored three arms (120° angle, 30 × 5 × 15 cm) for 5 min, with entry sequences and frequencies recorded. The spontaneous alternation rate (%) was calculated as (number of consecutive triple entries into different arms)/(total arm entries − 2) × 100, reflecting spatial working memory capacity.

### Histological examination

After 24 h of LPS injection, mice were anesthetized with intraperitoneal sodium pentobarbital (50 mg/kg), and blood was collected from the retro-orbital venous plexus using capillary tubes. Following centrifugation at 1000*g* and 4 °C for 10 min, serum was isolated for further biochemical analysis. The mice were then euthanized by cervical dislocation, and their spleen and brain tissues were removed and weighed. The organ index was then calculated. Brain tissues were fixed in 4% paraformaldehyde for 24 h. Next, brain tissues were paraffin-embedded and sectioned. 4 coronal sections per mouse containing the hippocampal CA1 and CA3 subregions (identified according to the mouse brain stereotaxic atlas) was selected for staining. Brain sections were subjected to hematoxylin–eosin (HE) staining to evaluate the effect of IVA on LPS-induced neuropathology. Furthermore, the brain sections were stained with toluidine blue to quantify the number of normal neurons in the hippocampal CA1/CA3 regions. In addition, immunofluorescence (IF) detected microglial marker ionized calcium-binding adapter molecule 1 (Iba1), while immunohistochemistry (IHC) measured phosphorylation of NF-κB p65, collectively evaluating IVA's protective potential against LPS-induced neuroinflammation. For each section, one representative field was randomly selected in the CA1 and CA3 subregions, respectively, for quantification, with scale bars applied to all images. Quantitative analyses were performed using ImageJ software, and all histological evaluations were conducted by investigators blinded to the treatment groups.

### Statistical analysis

The data are presented as mean ± standard deviation (SD) and analyzed using one-way ANOVA followed by Dunnett's multiple comparisons test and t-test in GraphPad Prism 9.5.1. Statistical significance was defined as p < 0.05.

## Results

### IVA exhibits a potent anti-neuroinflammatory activity in vitro

To assess the anti-neuroinflammatory properties of IVA (Fig. 1A), we investigated its inhibitory effect on the production of NO, a key pro-inflammatory biomarker, in LPS-activated microglia. As shown in Fig. 1B–E, IVA dose-dependently inhibited LPS-induced NO production in both BV2 cells and primary microglia without significantly affecting cell viability. Concurrently, qRT-PCR analysis revealed that IVA effectively reduced LPS-induced mRNA expression of iNOS (Fig. 1F), a critical inducible enzyme in NO synthesis. Additionally, we observed that LPS markedly upregulated the gene expression of COX-2, another inducible enzyme involved in inflammation induction, whereas IVA dose-dependently suppressed LPS-induced up-regulation of COX-2 mRNA (Fig. 1G). Furthermore, western blotting confirmed that IVA treatment significantly suppressed the LPS-induced abnormal elevation of iNOS and COX-2 proteins (Fig. 1H, I) in BV2 cells. These findings collectively demonstrated that IVA effectively counteracts LPS-induced neuroinflammation in microglial cells.

### IVA blocks LPS-induced NF-κB activation in BV2 cells

**Fig. 2:**
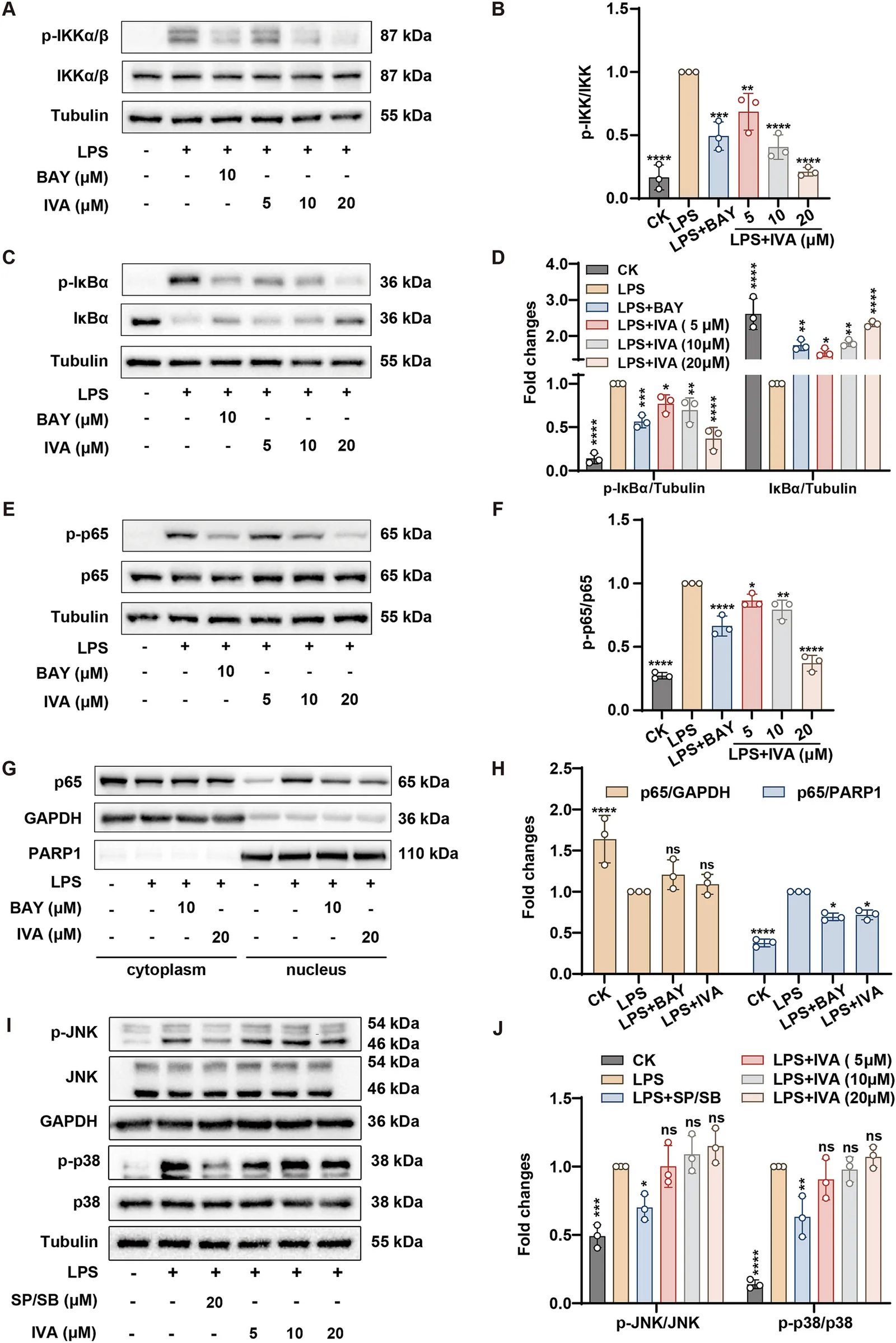
The effects of IVA on LPS-induced activation of NF-κB pathway in BV2 cells. Western blotting analysis of p-IKKα/β and total IKKα/β (**A**), p-IκBα and total IκBα (**C**), as well as p-p65 and total p65 (**E**) in BV2 cells following IVA treatment (2 h) and LPS stimulation (5 min). Densitometric quantification of p-IKKα/β, p-IκBα, IκBα, and p-p65, normalized to their total forms or Tubulin, respectively, is presented in (**B**, **D**, and **F**). For NF-κB p65 subcellular localization analysis, cytoplasmic and nuclear fractions from BV2 cells treated with IVA (2 h) and stimulated with LPS (30 min) were subjected to immunoblotting (**G**), with GAPDH and PARP1 serving as loading controls for cytoplasmic and nuclear fractions, respectively. Statistical results of p65 distribution, normalized to GAPDH or PARP1, are shown in (**H**). BV2 cells were treated with IVA (5, 10, and 20 μM), SP600125 (SP, 20 μM), or SB203580 (SB, 20 μM) for 2 h, followed by LPS stimulation for 30 min. **I** The phosphorylation levels of JNK and p38, as well as total protein levels of JNK, p38, GAPDH, and Tubulin were detected by western blotting (SP and SB were used as positive controls for p-JNK and p-p38, respectively). **J** The quantitative results of p-JNK and p-p38 normalized against total JNK and p38 are shown, respectively. *p < 0.05, **p < 0.01, ***p < 0.001, ****p < 0.0001, vs. LPS; ns means no significant; n = 3

Activation of NF-κB pathway by LPS triggers subsequent neuroinflammatory response [27]. To investigate whether IVA's anti-neuroinflammatory effects stem from NF-κB pathway inactivation, we performed western blotting analysis. Our findings revealed that IVA significantly suppressed LPS-induced phosphorylation of IKKα/β without affecting their total protein levels in BV2 cells (Fig. 2A and B). Moreover, IVA effectively counteracted LPS-induced phosphorylation and degradation of IκBα protein (Fig. 2C and D) and markedly reduced LPS-stimulated NF-κB p65 phosphorylation in BV2 cells (Fig. 2E and F). Subcellular fractionation experiments demonstrated that IVA substantially prevented LPS-induced nuclear translocation of p65 in BV2 cells (Fig. 2G and H). Notably, although IVA did not significantly inhibit LPS-induced JNK or p38 phosphorylation, a slight but non-significant upward trend in the p-JNK/JNK and p-p38/p38 ratios was observed at higher IVA concentrations (Fig. 2I and J). These findings suggest that the anti-neuroinflammatory effects of IVA are primarily associated with suppression of NF-κB signaling rather than a significant modulation of the JNK/p38 MAPK pathways under the present experimental conditions.

### IVA reduces the expression of NF-κB-targeted genes in LPS-stimulated BV2 cells

**Fig. 3:**
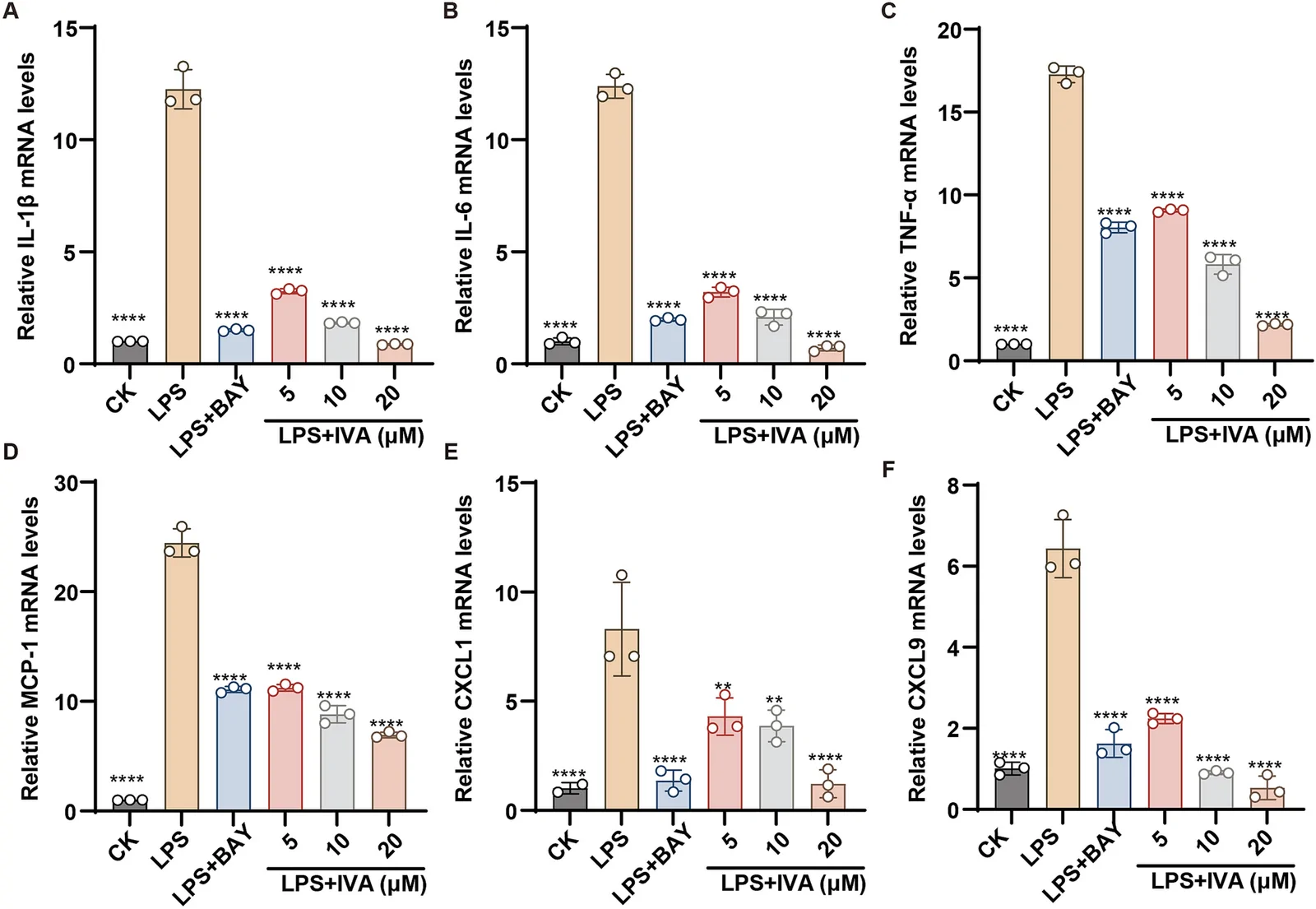
The effects of IVA on the mRNA expression of cytokines and chemokines in LPS-stimulated BV2 cells. BV2 cells were pretreated with IVA, BAY (positive control), or DMSO (vehicle control) for 1 h prior to LPS stimulation (12 h). The mRNA levels of pro-inflammatory cytokines IL-1β (**A**), IL-6 (**B**), and TNF-α (**C**), as well as chemokines MCP-1 (**D**), CXCL1 (**E**), and CXCL9 (**F**), were quantified by RT-qPCR. *p < 0.05, **p < 0.01, ***p < 0.001, ****p < 0.0001, vs. LPS, n = 3

The NF-κB signaling pathway regulates the expression of multiple pro-inflammatory cytokines [27]. Therefore, we employed qRT-PCR to assess the transcriptional levels of these cytokines. As demonstrated in Fig. 3A–C, LPS stimulation significantly upregulated the gene expression of pro-inflammatory cytokines, including IL-1β, IL-6, and TNF-α, which was dose-dependently inhibited by IVA. Additionally, various chemokines are also target genes of NF-κB. Our results revealed that LPS exposure enhanced mRNA expression of chemokines including monocyte chemotactic protein-1 (MCP-1), C-X-C motif chemokine (CXCL1), and CXCL9, while IVA treatment effectively suppressed LPS-induced expression of these chemokines in BV2 cells (Fig. 3D–3F). Concurrently, BAY, a known NF-κB pathway inhibitor, also substantially inhibited LPS-induced expression of both cytokines and chemokines. These findings collectively demonstrated that IVA exhibits a robust anti-neuroinflammatory activity by inhibiting the expression of NF-κB-targeted cytokines and chemokines.

### The α-methylene-γ-lactone moiety of IVA is required for its in vitro anti-neuroinflammatory activity

**Fig. 4:**
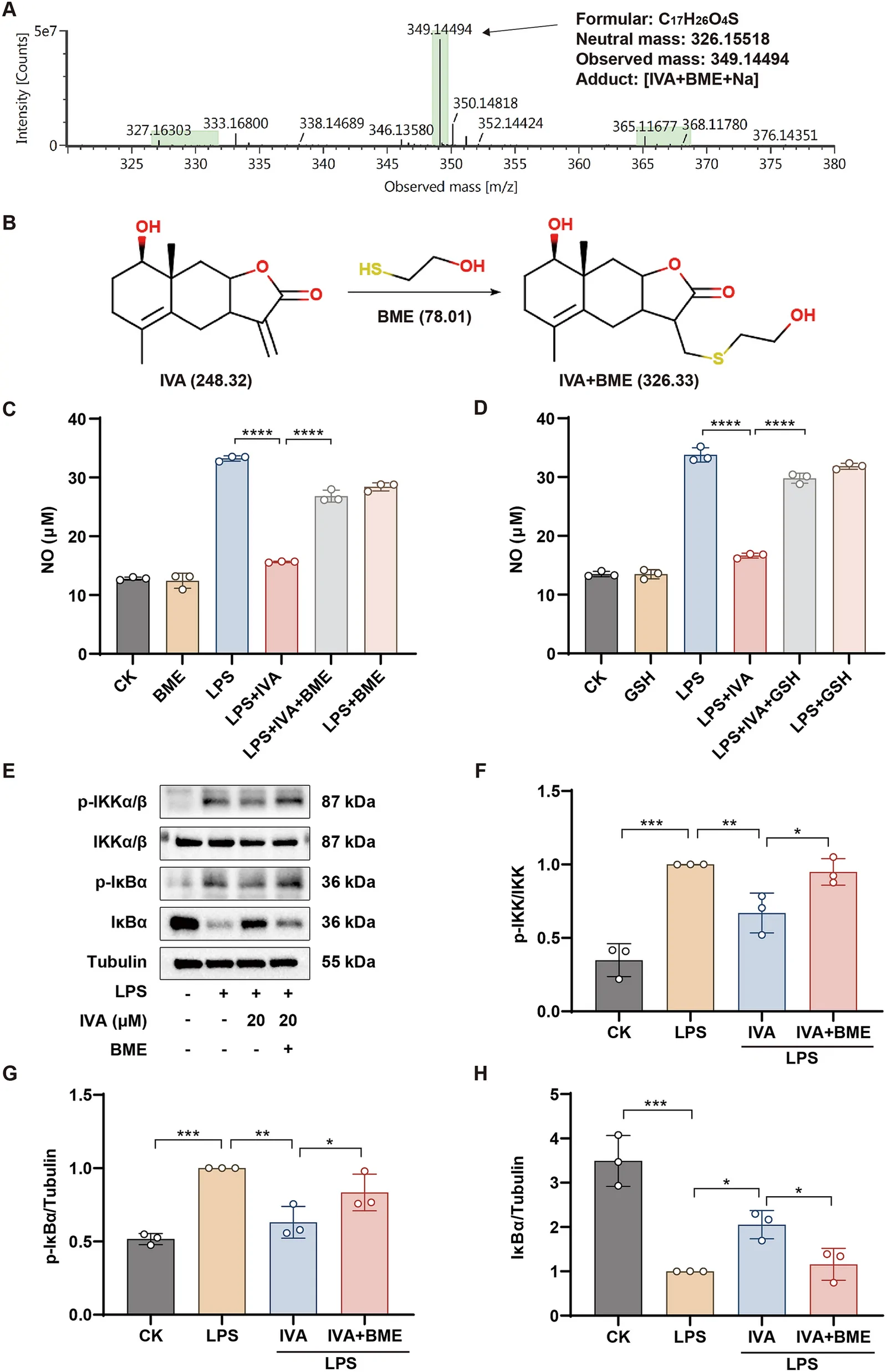
The covalent modification properties and functional implications of IVA. **A** The IVA-BME adduct was identified by LC–MS analysis. **B** Proposed mechanism for the Michael addition reaction between IVA and BME. **C**, **D** BV2 cells were stimulated with LPS for 18 h in the presence of IVA (20 μM), BME-preincubated IVA, or GSH-pretreated IVA, followed by measurement of NO production using the Griess assay. The concentration of BME and GSH used in this assay was 100 μM. (**E**) Representative western blots of p-IKKα/β, p-IκBα, and total IκBα in BV2 cells treated with IVA and stimulated with LPS. **F**–**H** Densitometric quantification of p-IKKα/β, p-IκBα, and IκBα protein levels, normalized to their respective total forms or Tubulin, respectively. *p < 0.05, **p < 0.01, ***p < 0.001, ****p < 0.0001, n = 3

The α,γ-unsaturated lactone moiety in IVA functions as a Michael acceptor, conferring electrophilic reactivity that facilitates covalent binding to protein cysteine residues via Michael addition. To mechanistically characterize this interaction, LC–MS analysis using BME as a thiol-containing surrogate for protein cysteine residues revealed the formation of a specific [IVA + BME + Na] adduct (m/z 349.14) (Fig. 4A), demonstrating a selective thiol covalent modification (Fig. 4B). Functional validation further confirmed that pre-incubation with BME or another thiol donor glutathione (GSH) completely abrogated IVA's inhibitory effects on NO production in LPS-stimulated BV2 cells (Fig. 4C and D). Furthermore, the covalent modification of IVA by BME completely abrogated its inhibitory effects on LPS-induced phosphorylation of IKKα/β and IκBα, as well as subsequent IκBα degradation in BV2 cells (Fig. 4E–H). Therefore, the α-methylene-γ-lactone structural motif in IVA constitutes the essential pharmacophore responsible for its anti-neuroinflammatory efficacy in vitro.

### UBE2L3 is identified as the anti-neuroinflammatory target of IVA

**Fig. 5:**
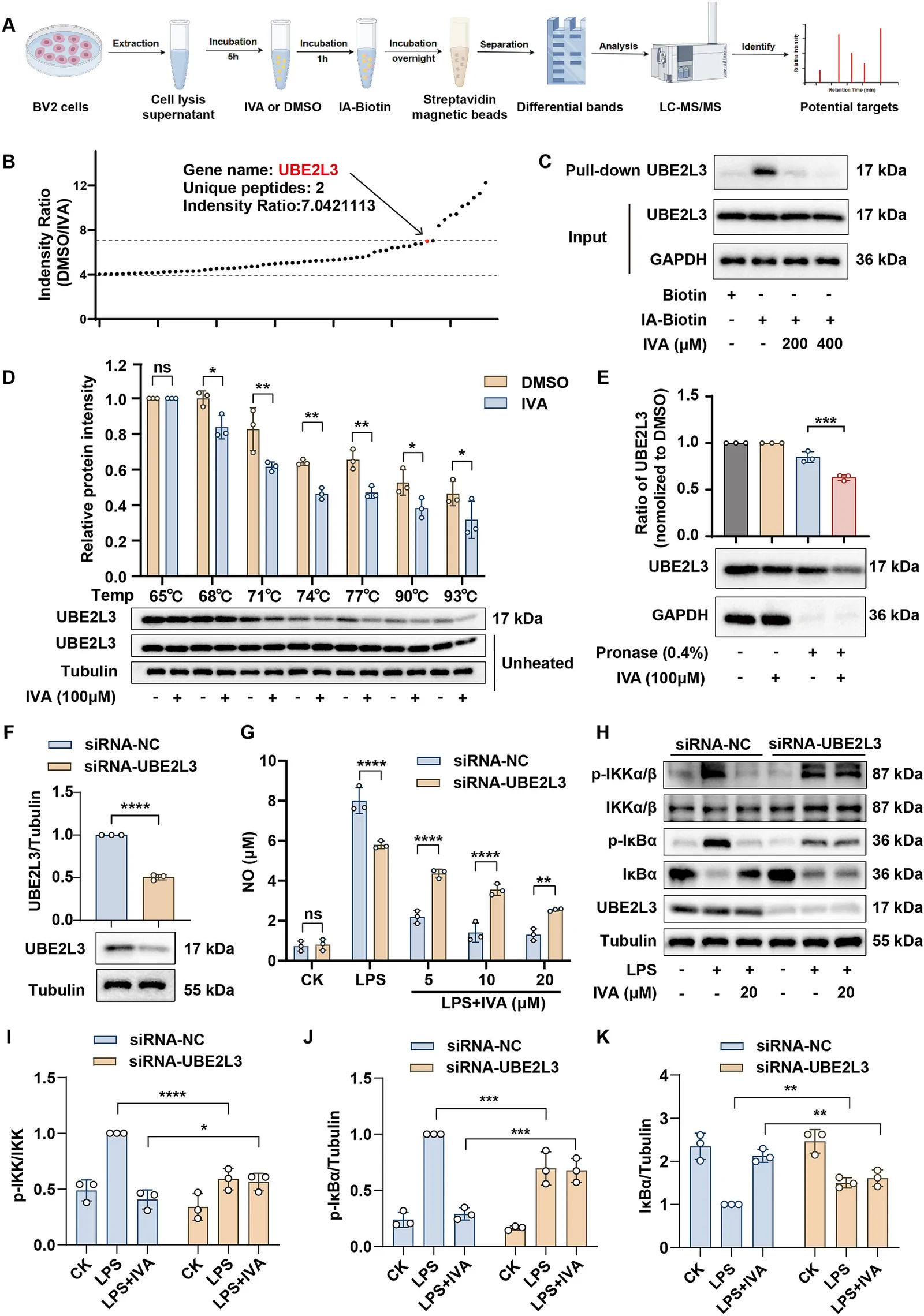
UBE2L3 serves as the anti-neuroinflammatory target of IVA. **A** Schematic workflow for profiling potential IVA-binding proteins using Biotin-IA probe. **B** Chemical proteomic screening of IVA-interacting proteins in BV2 cells. **C** Pull-down assay confirming direct binding between IVA and UBE2L3. Interaction was further validated by CETSA (**D**) and DARTS (**E**) assays, with GAPDH or Tubulin serving as internal controls. The CETSA analysis reflects UBE2L3 thermal stabilization between 65 °C and 93 °C, with band intensity normalized to the value at 65 °C (**D**). For DARTS, UBE2L3 levels were normalized to pronase-untreated controls (**E**). **F** Western blotting analysis of UBE2L3 knockdown efficiency after 48 h siRNA transfection. **G** Impact of IVA on LPS-induced NO production in UBE2L3-knockdown versus negative control (NC) BV2 cells. **H** UBE2L3 knockdown reversed the inhibitory effect of IVA on NF-κB pathway activation. **I**–**K** Densitometric quantification of p-IKKα/β, p-IκBα, and IκBα normalized to total protein or Tubulin, respectively. *p < 0.05, **p < 0.01, ***p < 0.001, ****p < 0.0001, n = 3

Given IVA's capacity for covalent binding to nucleophilic cysteines, we utilized a cysteine-reactive Biotin-IA probe combined with competitive pull-down to identify its protein targets in BV2 cells (Fig. 5A). As shown in Fig. 5B, protein targets with fold changes greater than 4 and unique peptide counts exceeding 2 were identified. Among them, UBE2L3, a ubiquitin conjugating enzyme, with a fold change of 7.04, was identified as a potential target for IVA's anti-neuroinflammatory activity (Fig. 5B). Furthermore, pull-down assay confirmed that the abundance of UBE2L3 was significantly reduced in the presence of IVA compared to the Biotin-IA-only group (Fig. 5C). Moreover, CETSA and DARTS analyses demonstrated that UBE2L3 protein exhibited lower thermal stability and resistance to protease digestion in the presence of IVA (Fig. 5D and 5E). Notably, the inhibitory effect of IVA on LPS-induced NO production was significantly attenuated upon UBE2L3 knockdown (Fig. 5F and G). Meanwhile, UBE2L3 knockdown also markedly attenuated IVA-induced inhibition on phosphorylation of IKKα/β and IκBα as well as degradation of IκBα in LPS-treated BV2 cells (Fig. 5H–K). Therefore, these results collectively demonstrated that UBE2L3 serves as a critical functional target mediating IVA's anti-neuroinflammatory action‌.

### IVA binds to UBE2L3 by covalently modifying Cys86

**Fig. 6:**
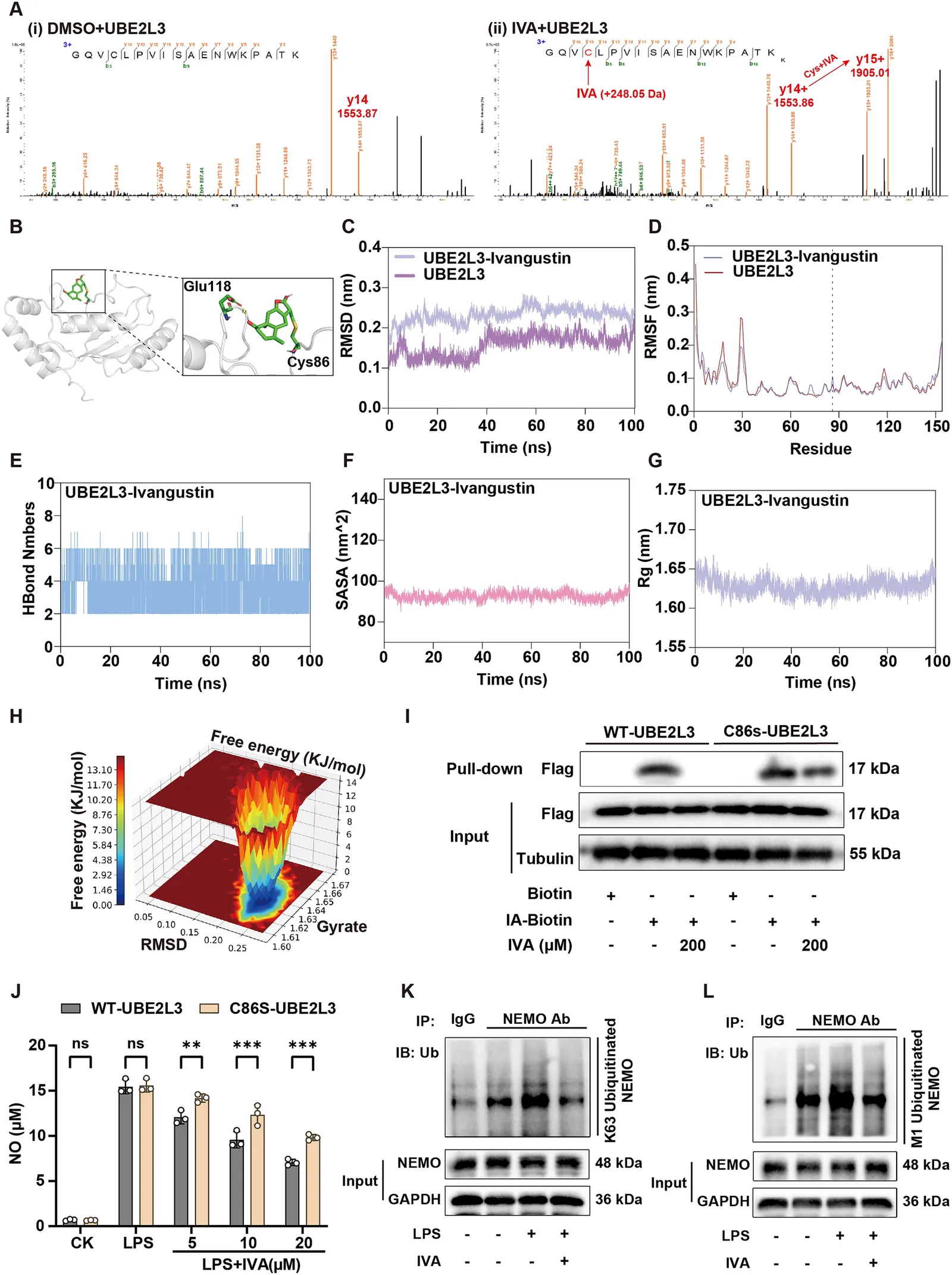
IVA covalently interacts with Cys86 residue in UBE2L3 to inhibit its function. **A** LC–MS/MS analysis of the recombinant human UBE2L3 protein incubated with DMSO (left panel) or IVA (right panel). The diagnostic peptide GQVCLPVISAENWKPATK (Cys86 in red) shows a + 248.05 Da mass shift (y14⁺: m/z 1553.87 → y15⁺: m/z 1905.01) in the right spectrum, confirming covalent modification of Cys86 by IVA. **B** 3D covalent binding models between IVA and UBE2L3 are shown. RMSD values (**C**), RMSF values (**D**), HBonds values (**E**), SASA values (**F**), Rg values (**G**), and Free energy landscape (**H**) of IVA-UBE2L3 complex detected using molecular dynamic simulation. (**I**) Pull-down assay comparing IVA binding to WT-UBE2L3 versus C86S-UBE2L3 in HEK293T cells. (**J**) In BV2 cells following endogenous UBE2L3 knockdown, overexpression of WT-UBE2L3 or C86S-UBE2L3, followed by assessment of IVA's inhibitory effect on LPS-induced NO production. **K**, **L** After being treated with IVA for 2 h, BV2 cells were incubated with LPS for additional 5 min. Then, co-IP coupled with western blotting was applied to examine the K63 and M1 ubiquitination of NEMO

The α, γ-unsaturated lactone group in IVA functions as a Michael acceptor, enabling covalent conjugation with cysteine residues, we therefore performed an LC–MS/MS assay to determine the binding site of UBE2L3 covalently modified by IVA. As shown in Fig. 6A, a diagnostic peptide (GQVCLPVISAENWKPATK) that exhibited a + 248.05 Da mass shift following IVA incubation was localized to y14 +  → y15 + fragment ions, consistent with IVA's molecular weight and confirming the covalent modification of the catalytic Cys86 in UBE2L3 (Fig. 6A). Molecular docking demonstrated that IVA's C11 = C13 double bond engages in Michael addition with Cys86's thiol group, with a docking score of − 5.8 kcal/mol and a C–S distance of 2.1 Å, while the stabilization of the interaction is mediated by a hydrogen bond between the hydroxyl of IVA and the carbonyl of Glu118 (Fig. 6B). MD simulations revealed that the IVA-UBE2L3 complex exhibited increased RMSD mean values and enhanced fluctuations, indicating ligand-induced receptor conformational rearrangement (Fig. 6C). RMSF analysis demonstrated heightened dynamics around residue Cys86, supporting the induced-fit mechanism (Fig. 6D). The complex stably maintained 2–7 hydrogen bonds (Fig. 6E), with saturated hydrophobic interactions (stable SASA, Fig. 6F) and localized conformational changes (stable Rg) (Fig. 6G). Free energy landscape analysis identified a single dominant conformational state (ΔG≈13.1 kJ/mol, Fig. 6H), which together with persistent hydrogen bonds and localized flexibility regulation, collectively confirmed the complex's high affinity and specificity.

To further validate the functional importance of Cys86, we performed point mutation in two complementary cell systems. Pull-down assays in HEK293T cells revealed that the C86S mutation significantly reduced IVA binding to UBE2L3 compared to wild-type (Fig. 6I). In BV2 cells, WT-UBE2L3 overexpression significantly restored IVA's inhibitory effect on LPS-induced NO production in the UBE2L3 knockdown background, whereas C86S-UBE2L3 overexpression failed to achieve a comparable effect (Fig. 6J). These results collectively demonstrate that Cys86 is the critical residue for both IVA binding and its anti-neuroinflammatory activity.

As a ubiquitin-conjugating enzyme, UBE2L3 mediates the K63 and M1 polyubiquitination of NEMO, a critical component of IKK complex assembly during pro-inflammatory signaling [28, 29]. As expected, LPS stimulation markedly enhanced K63 and M1 ubiquitination of NEMO in BV2 cells, which were substantially attenuated by IVA treatment (Fig. 6K and L). Overall, these results indicated that IVA covalently binds to the catalytic Cys86 of UBE2L3 via a Michael addition, thereby suppressing its ubiquitin-conjugating enzyme activity.

### Up-regulated UBE2L3 exhibits a positive correlation with neuroinflammation

**Fig. 7:**
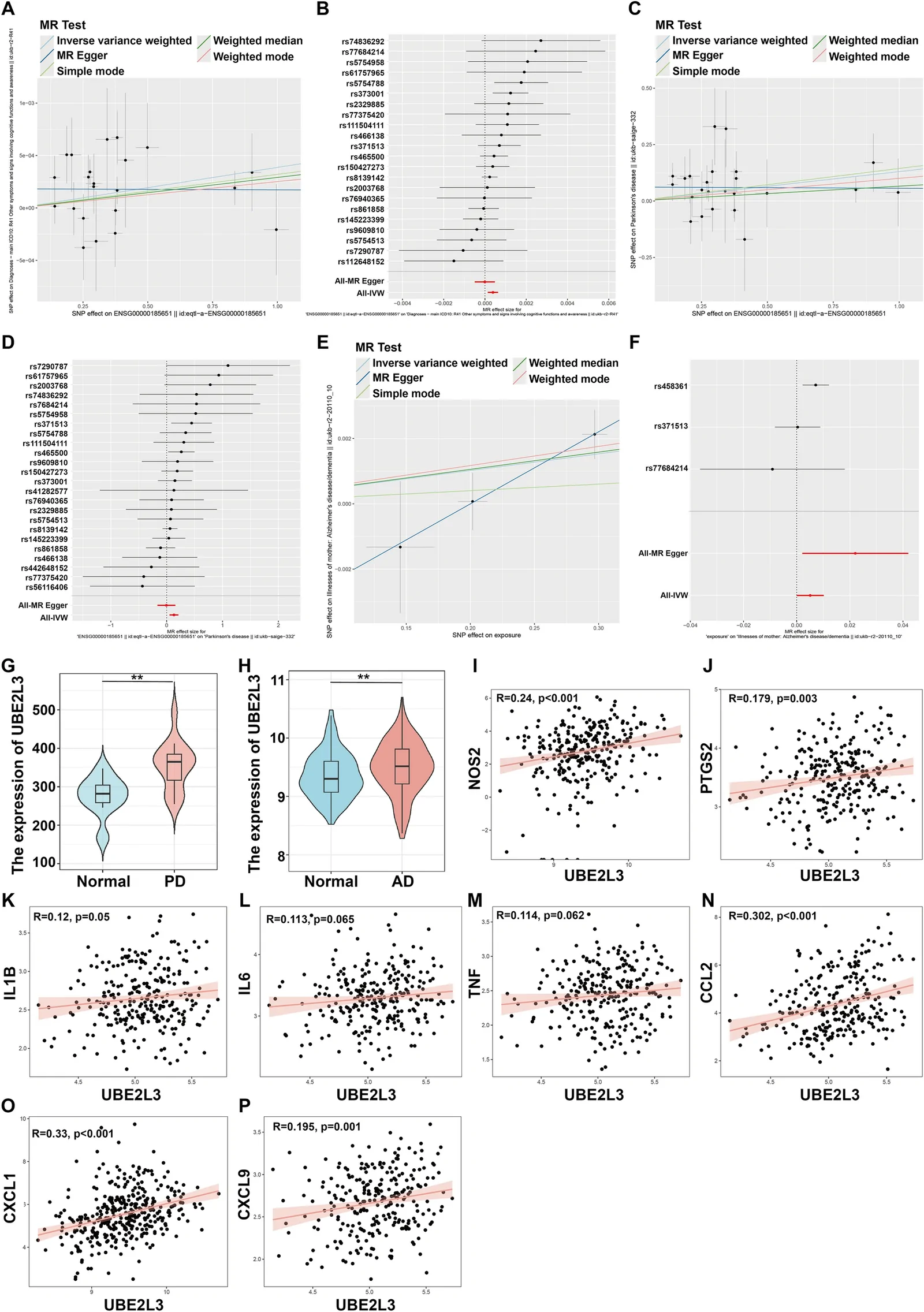
UBE2L3 is upregulated in neurodegenerative diseases and positively correlated with neuroinflammation. Scatter plot of associations of SNPs with UBE2L3 expression and the risk of PD (**A**), cognitive impairment (**C**), and AD (**E**) based on IVW, MR-Egger, weighted median, simple mode, and weighted mode, respectively. Forest plot of associations of UBE2L3 SNPs and the prognosis of PD (**B**), cognitive impairment (**D**), and AD (**F**) based on MR-Egger and IVW methods. **G**, **H** The transcriptional expression levels of UBE2L3 in brain tissues of patients with PD and AD were analyzed from the GEO databases GSE7621 and GSE15222, respectively. **G**–**P** UBE2L3-microglial inflammatory mediator relationship. Each dot represents the gene expression level from an individual patient as recorded in the databases of GSE15222 and GSE11855

To further elucidate the role of UBE2L3 in neuroinflammation, we conducted bioinformatic analyses to explore its correlation with cognitive impairment, AD, and PD. MR analysis revealed significant causal associations between genetically predicted higher UBE2L3 expression and increased risk of both PD and cognitive impairment. Using IVW as the primary method, each unit increase in genetically predicted UBE2L3 expression was associated with a 13.9% higher risk of PD (OR = 1.139, 95% CI 1.052–1.234, p = 0.0014; FDR-adjusted p = 0.017) and a significant increase in the risk of cognitive impairment (OR = 1.0004, 95% CI 1.0001–1.0006, p = 0.0027; FDR-adjusted p = 0.027) (Fig. 7A–D). Sensitivity analyses supported the robustness of these findings: Cochran's Q test revealed no significant heterogeneity for PD (Q = 23.84, p = 0.425) or cognitive impairment (Q = 18.62, p = 0.196); MR-Egger intercept tests showed no evidence of horizontal pleiotropy for PD (intercept = -0.012, p = 0.072) or cognitive impairment (intercept = 0.003, p = 0.085); and leave-one-out analysis confirmed that the results were not driven by any single influential SNP (Supplementary Figs. 2A-B). The complementary MR methods (weighted median, MR-Egger, simple mode, and weighted mode) showed consistent effect directions, further supporting the causal associations (Fig. 7A and C).

For AD, the IVW analysis showed a weak association that reached only nominal significance (OR = 1.005, 95% CI 1.000–1.010, p = 0.050; FDR-adjusted p = 0.676), which did not survive multiple testing correction (Fig. 7E–F). Sensitivity analyses indicated no significant heterogeneity (Q = 1.23, p = 0.234) or horizontal pleiotropy (intercept = 0.002, p = 0.338). Although the effect direction was consistent with that observed for PD and cognitive impairment, the limited statistical power (only 3 SNPs from the AD GWAS with strict LD threshold) and the lack of significance after correction suggest that the evidence for a causal role of UBE2L3 in AD remains preliminary. Leave-one-out analysis further confirmed the instability of this finding: with only 3 SNPs available, removal of a single SNP shifted the estimate toward the null (Supplementary Fig. 2C), underscoring the need for larger AD GWAS to validate this tentative signal.

Analysis of GEO database further revealed that UBE2L3 mRNA expression was significantly upregulated in patients with neuroinflammatory diseases such as AD and PD compared to healthy controls (Figs. 7G and H). Subsequently, we found that UBE2L3 overexpression exhibited widespread positive correlations with key neuroinflammatory markers (Fig. 7I–P). For example, up-regulated UBE2L3 was strongly correlated with both microglia/macrophage activation biomarkers (iNOS/NOS2: R = 0.24, p < 0.001, Fig. 7I; COX-2/PTGS2: R = 0.179, p = 0.003, Fig. 7J) and key chemokines regulating immune cell recruitment (C–C motif chemokine 2 (CCL2)/MCP-1: R = 0.302, p < 0.001, Fig. 7N; CXCL1: R = 0.33, p < 0.01, Fig. 7O; CXCL9: R = 0.195, p = 0.001, Fig. 7P). While IL-1β showed a marginally significant association (R = 0.12, p = 0.05, Fig. 7K), IL-6 and TNF-α exhibited positive trends that did not reach statistical significance (Fig. 7L and M). Collectively, these bioinformatics analyses suggest that elevated UBE2L3 expression is positively associated with neuroinflammation and may serve as a potential risk factor for neurodegenerative diseases, supporting its relevance as a therapeutic target for further investigation.

### IVA alleviates LPS-induced cognitive impairment in mice

**Fig. 8:**
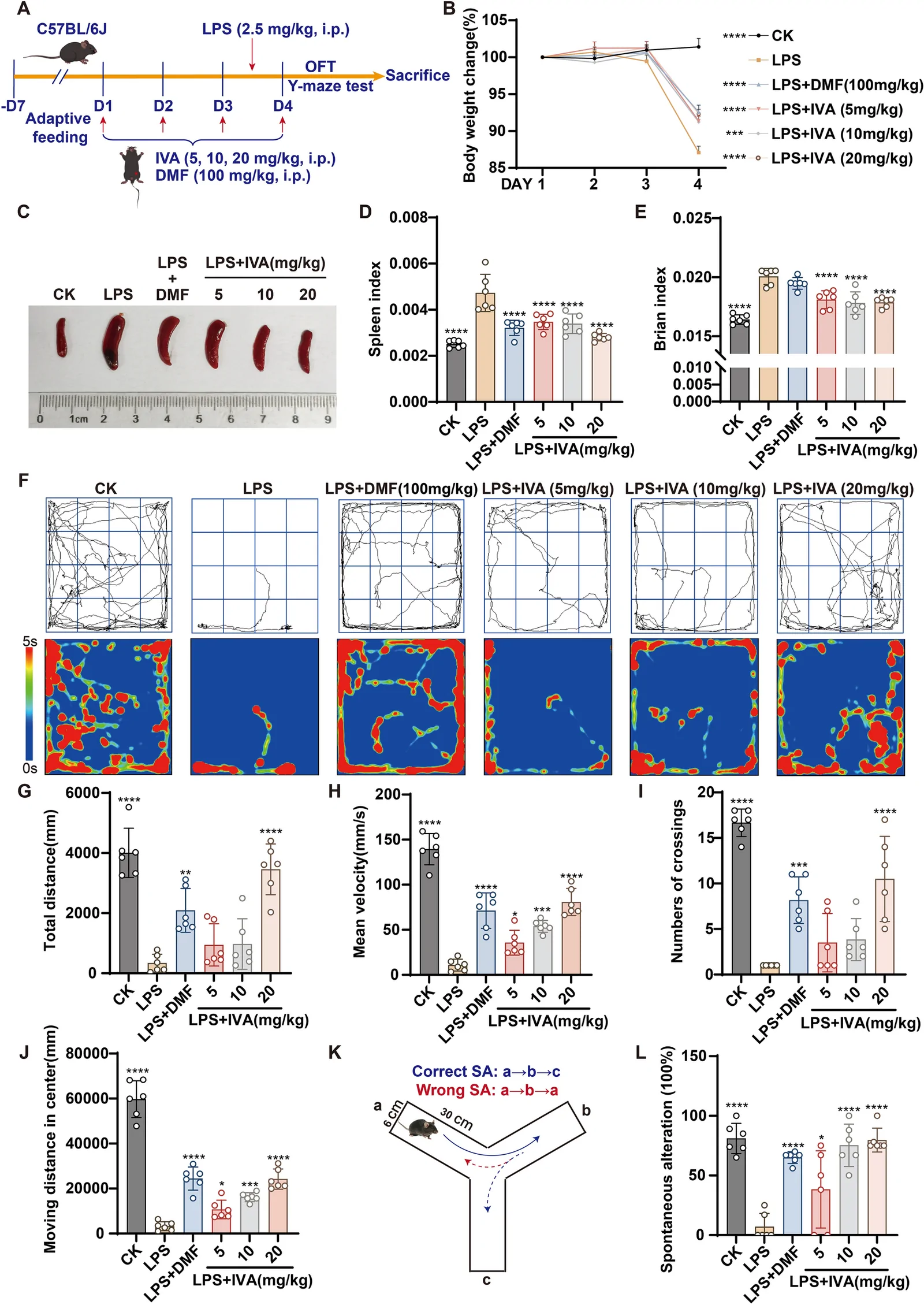
The improvement effects of IVA on LPS-induced cognitive dysfunction in mice. **A** Schematic illustration of the experimental protocol. **B** Body weight variations in mice during experiment. **C** Representative spleens from each experimental group. **D** Spleen index (spleen weight/body weight). **E** Brain tissue index (brain weight/body weight). Behavioral alterations were assessed using the open field test. **F** Representative activity trajectory plots and heatmaps for each group. Subsequently, the total travel distance (**G**) mean velocity (**H**) frequency of entries into the central zone (**I**), and movement distance (**J**) within this area were quantified. **K** Schematic representation of the Y-maze experiment. **L** The spontaneous alternation rate in each arm of the Y-maze was subsequently calculated for each group. *p < 0.05, **p < 0.01, ***p < 0.001, ****p < 0.0001, vs. LPS, n = 6

To further investigate the anti-neuroinflammatory effects of IVA, we established a mouse model of acute cognitive dysfunction by intraperitoneal injection of LPS and evaluated the neuroprotective effect of IVA through behavioral assays (Fig. 8A). As shown in Fig. 8B, LPS challenge significantly reduced mouse body weight, whereas IVA and DMF treatments effectively inhibited LPS-induced weight loss. Furthermore, IVA administration also notably suppressed LPS-induced increases in spleen (Fig. 8C–D) and brain tissue coefficients (Fig. 8E). Subsequent open-field test revealed that IVA therapy not only significantly ameliorated LPS-induced reductions in total movement distance and mean velocity (Fig. 8F–H), but also effectively mitigated LPS-induced decreases in the frequency of entries into the central zone and movement distance within this area (Fig. 8F and I, J). Crucially, Y-maze testing demonstrated that LPS exposure caused pronounced short-term memory impairment in mice, evidenced by a significant decline in spontaneous alternation rate, which was markedly improved by IVA treatment (Fig. 8K and L). Although LPS exposure also reduced total arm entries, indicating decreased locomotor activity, the total arm entries in IVA-treated groups showed an increasing trend without statistical significance compared with the LPS group (Supplementary Fig. 3). This suggests that the improvement in alternation rate cannot be fully attributed to locomotor recovery. Taken together, IVA is found to reduce LPS-induced cognitive impairment in mice.

### IVA mitigates LPS-induced neuroinflammation in mice

**Fig. 9:**
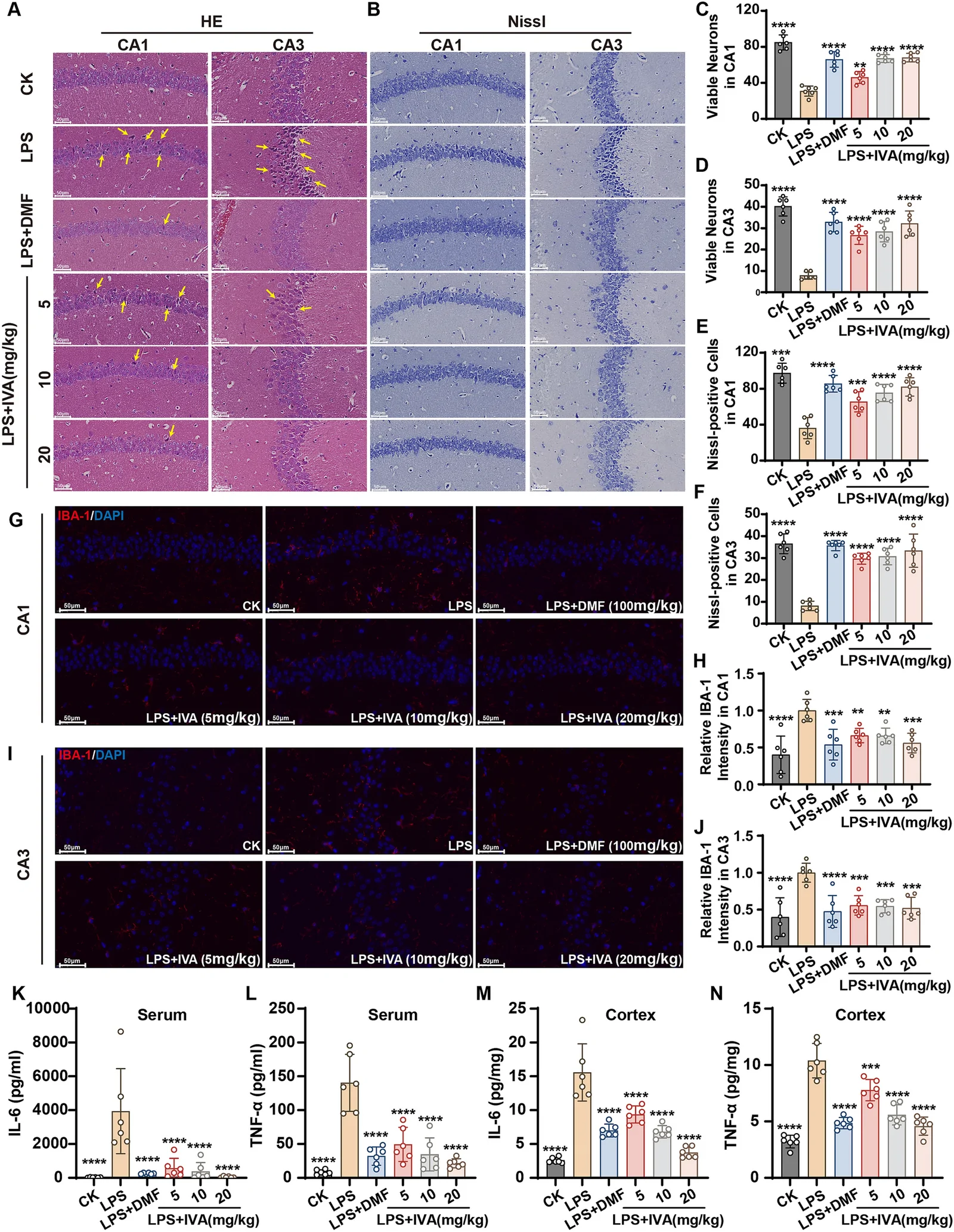
The attenuation effects of IVA on neuroinflammation in LPS-stimulated mice. **A** HE staining analysis demonstrated pathological alterations in hippocampal CA1 and CA3 subfields. Yellow arrows highlight regions exhibiting increased basophilic staining intensity. **C**, **D** Viable neuron counts were systematically quantified in both hippocampal subfields. **B** Nissl staining revealed neuronal density and structural changes in CA1-CA3 regions. Damaged neurons exhibited features such as cell body rupture, nuclear shrinkage, and condensation, appearing as intensely stained aggregates or diffuse dark staining. **E**, **F** Quantitative analysis of Nissl-positive neurons was performed for both subfields. **G**, **I** IF staining analyzed Iba1 expression (red) in hippocampal CA1 and CA3 regions, with DAPI counterstaining nuclei (blue). **H**, **J** Relative expression levels of Iba1 were quantitatively assessed in these regions. **K**–**N** ELISA analysis of IL-6 and TNF-α levels in serum (**K**, **L**) and cortex (**M**, **N**). *p < 0.05, **p < 0.01, ***p < 0.001, ****p < 0.0001, vs. LPS, n = 6

To further evaluate the pharmacological effect of IVA against LPS-induced neuroinflammation and cognitive dysfunction, we conducted histological analysis of hippocampal neuronal injury using HE and Nissl staining. As demonstrated in Fig. 9A, 9C and 9D, LPS exposure resulted in a significant reduction in neuronal density within the hippocampal CA1 and CA3 regions of mice, accompanied by characteristic morphological alterations including neuronal shrinkage, degeneration, necrosis, irregularly condensed nucleoli, and intensified basophilic staining. Notably, both IVA and DMF treatment effectively mitigated these LPS-induced neuronal abnormalities in the CA1 and CA3 subfields. Simultaneously, Nissl staining showed that LPS injection significantly increased damaged neurons in the hippocampus, featuring shrunken and condensed nuclei with intensely stained clumps or diffuse deep staining. In contrast, IVA and DMF treatment significantly increased the number of Nissl-positive neurons in the hippocampus of LPS-exposed mice (Fig. 9B, E and F). Subsequently, IF analysis revealed that IVA and DMF treatment significantly suppressed LPS-induced elevation of the microglial activation biomarker Iba1 in hippocampal CA1 and CA3 regions (Fig. 9G–J). We next measured pro-inflammatory cytokine levels in serum and cortical homogenates by ELISA to assess systemic and central inflammatory responses. As shown in Fig. 9K–N, LPS challenge significantly elevated TNF-α and IL-6 levels in both serum and cortex, which were markedly reduced by IVA treatment.

**Fig. 10:**
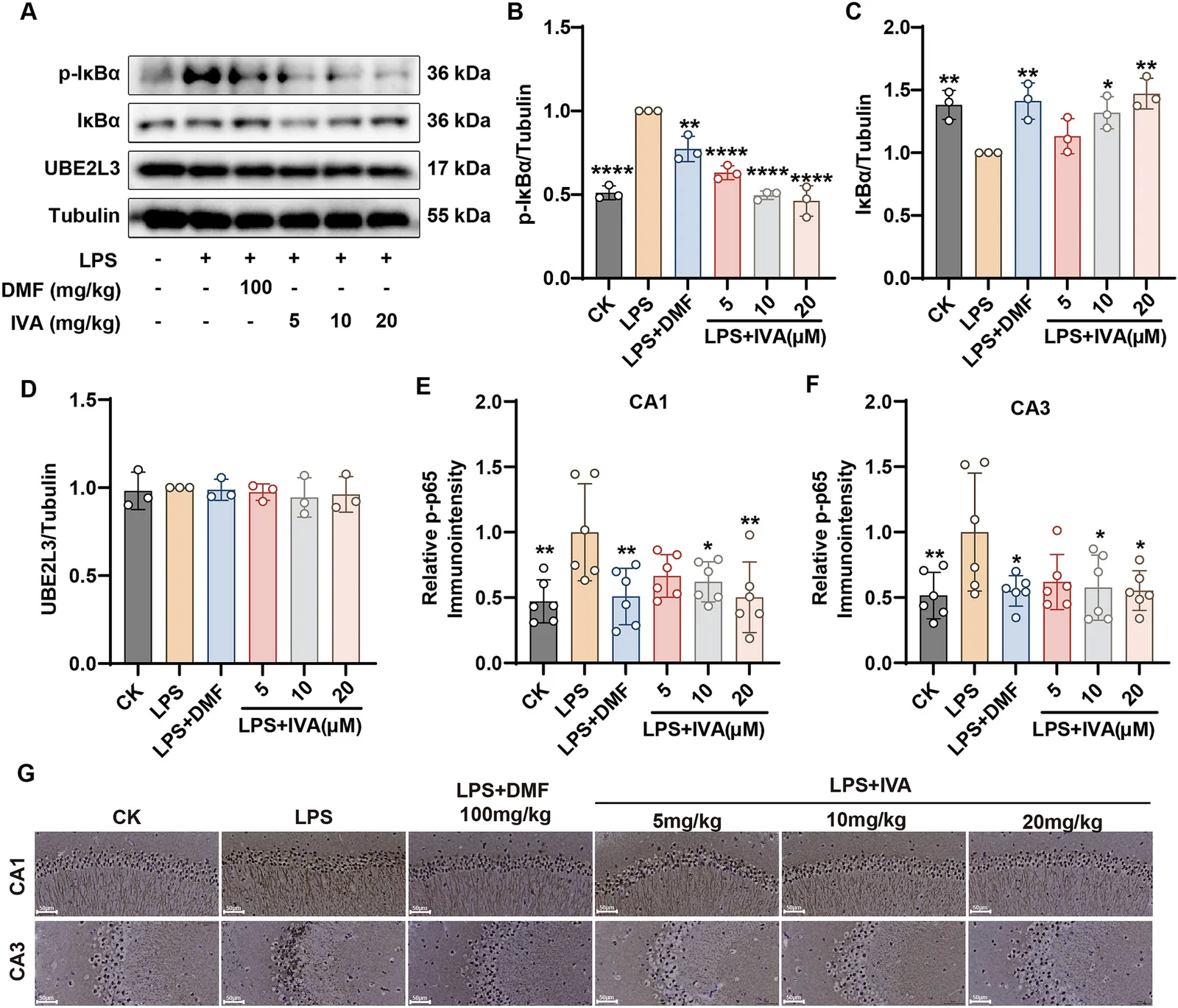
Effects of IVA on UBE2L3/NF-κB signaling in mouse brain. Cortical tissues were collected 24 h after LPS injection. **A** Representative Western blots of p-IκBα, IκBα, UBE2L3, and Tubulin in cortical lysates. **B**–**D** Densitometric quantification of p-IκBα, IκBα, and UBE2L3 protein levels normalized to Tubulin (n = 3 per group). **E**, **F** Representative images of p-p65 immunohistochemical staining in hippocampal CA1 and CA3 regions. **G** Quantification of p65 phosphorylation levels (n = 6 per group). Data are presented as mean ± SD. *p < 0.05, **p < 0.01, ***p < 0.001 vs. LPS

To determine whether the UBE2L3/NF-κB axis is engaged in vivo, we performed Western blot analysis of cortical lysates and IHC staining of hippocampal tissues. Western blot analysis revealed that LPS-induced IκBα phosphorylation and degradation were significantly attenuated by IVA treatment, whereas UBE2L3 protein levels remained unchanged across all groups (Fig. 10A–D). In addition, IHC analysis demonstrated that LPS-induced p65 phosphorylation in hippocampal tissues was also effectively reversed by IVA treatment (Fig. 10E–G). These in vivo data align with our in vitro findings, further supporting that IVA suppresses neuroinflammation through modulation of the UBE2L3/NF-κB axis.

## Discussion

Existing evidence demonstrates that abnormally activated microglia in the brain significantly exacerbate neuroinflammation through upregulating pro-inflammatory cytokines, recruiting peripheral immune cell infiltration, and disrupting the blood–brain barrier, and ultimately causing neuronal dysfunction and cognitive decline [6, 8]. Consequently, microglia-mediated neuroinflammation has emerged as a central pathological mechanism in cognitive impairment and neurodegenerative diseases, highlighting the clinical importance of developing anti-neuroinflammatory therapeutic strategies [6, 8]. Numerous recent studies have demonstrated that multiple TCM herbs and their derived natural products can effectively alleviate the pathological progression of neurodegenerative diseases via inhibiting microglial inflammatory phenotypes [30]. Our study represents the first identification of IVA, a sesquiterpene lactone isolated from *Inula japonica* Thunb., as a potent inhibitor of LPS-induced activation in BV2 murine microglia, as evidenced by reduced production of NO and mRNA levels of pro-inflammatory cytokines (IL-1β, IL-6, and TNF-α) and chemokines (MCP-1, CXCL1, and CXCL9). Notably, excessive NO production induces oxidative stress, triggering both inflammation and neuronal death [31], while elevated pro-inflammatory cytokines and chemokines create a self-sustaining inflammatory cycle that exacerbates neuronal damage [32], thereby processing critically implicated in AD and PD. Therefore, IVA represents a promising protective candidate for neuroinflammation-related cognitive disorders through its suppression of microglial hyperactivity.

In the LPS-induced neuroinflammation mouse model, our study demonstrated that IVA treatment effectively ameliorated LPS-induced spatial memory impairment and motor dysfunction. Both HE and Nissl staining results further showed that IVA administration significantly alleviated LPS-induced neuronal injury and reduced neuron count. Additionally, IF staining confirmed that IVA notably inhibited the LPS-induced increase in expression of Iba1, a key biomarker of microglial activation [33]. These in vivo findings were consistent with the results obtained from the in vitro microglial experiment, collectively suggesting that IVA's improvement of neuroinflammation and cognitive dysfunction is closely linked to its inhibition of microglial activation. Moreover, we observed that DMF, an approved therapeutic for the neuroinflammatory condition multiple sclerosis [20], also significantly mitigated LPS-induced neuroinflammation and cognitive dysfunction in mice at a dose of 100 mg/kg. Notably, ivangustin at 20 mg/kg produced protective effects comparable to those of DMF at 100 mg/kg in this model, providing a reference for its anti-neuroinflammatory potential and supporting its further preclinical investigation. However, given that this acute LPS model does not fully recapitulate the complex pathophysiology of chronic neurodegenerative diseases such as AD or PD, the protective effects of IVA observed here warrant further validation in disease-specific models.

Generally, neuroinflammation is strongly linked to overactivation of pro-inflammatory signaling pathways, including NF-κB and MAPKs, in microglia [7, 8]. As a potent Toll-like receptor 4 (TLR4) agonist, LPS triggers activation of these pathways [34]. Our study demonstrated that IVA effectively inhibited LPS-induced phosphorylation of IKKα/β and its substrate IκBα in BV2 cells. IκBα phosphorylation initiates its rapid degradation via the ubiquitin–proteasome system, relieving its inhibitory effect on NF-κB and promoting NF-κB phosphorylation and nuclear translocation [35]. Western blot analysis confirmed that IVA treatment significantly suppressed LPS-induced IκBα degradation, NF-κB p65 phosphorylation, and p65 nuclear translocation in BV2 cells. RT-qPCR further validated IVA's inhibitory effect on the mRNA expression of NF-κB-target genes, further substantiating its inhibition of the NF-κB pathway. Notably, while LPS exposure markedly increased JNK and p38 MAPK phosphorylation [25, 26], IVA had minimal effect on these pathways. Although a modest upward trend in p-JNK/JNK and p-p38/p38 ratios was observed at higher IVA concentrations, these changes were not statistically significant. Therefore, the present data do not support a significant inhibitory effect of IVA on JNK/p38 MAPK signaling under the current experimental conditions. Nevertheless, given the observed trend, a potential interaction between IVA and MAPK signaling cannot be completely excluded and warrants further investigation. Collectively, these findings suggest that IVA-mediated anti-neuroinflammatory effects are primarily mediated through NF-κB pathway inhibition.

The deep development of natural products is frequently hindered by inadequate knowledge of their direct targets. Target identification therefore represents a pivotal step in advancing natural product development [36, 37]. To elucidate the anti-neuroinflammatory target of IVA, we initially analyzed its chemical properties. The α-methylene-γ-lactone moiety within IVA's structure can function as an electrophilic group, undergoing Michael addition with the thiol group of target protein cysteine. Using the cysteine mimic BME, LC/MS analysis demonstrated that IVA forms an adduct with BME, thereby confirming the electrophilic activity of this moiety. Leveraging this property, we utilized the biotinylated cysteine-reactive probe Biotin-IA, with IVA as a competitor, to capture its covalently bound target proteins. LC–MS/MS combined with Pulldown analysis identified the ubiquitin-conjugating enzyme UBE2L3 as a potential target of IVA, which was subsequently validated by CETSA and DARTS. Furthermore, previous studies revealed that UBE2L3 participates in NF-κB signaling activation by promoting linear ubiquitination of NEMO [38], indicating UBE2L3 as a candidate target for NF-κB inhibition [21]. Bioinformatics analysis further corroborated that UBE2L3 expression is significantly elevated in patients with AD and PD, with its high expression exhibiting a positive correlation not only with cognitive impairment and PD risk but also with the levels of neuroinflammatory-related factors. Additionally, UBE2L3 polymorphisms were found to promote plasma cell development by enhancing NF-κB activation, thereby increasing susceptibility to multiple autoimmune diseases [28, 39]. Most notably, UBE2L3 knockdown markedly attenuated IVA-mediated NF-κB inhibition and anti-neuroinflammatory activity. However, these bioinformatics findings should be interpreted with caution, as the eQTL data were derived from peripheral blood rather than brain tissue, and warrant further validation in brain-derived datasets and disease-specific models. Collectively, these findings suggest that UBE2L3 is a primary candidate target for IVA's anti-neuroinflammatory activity. Furthermore, given UBE2L3's established role in autoimmune pathogenesis, IVA may also represent a promising protective candidate for autoimmune disorders.

UBE2L3, a pivotal regulator of NF-κB signaling pathway, plays a critical role in the pathogenesis of various inflammatory and autoimmune diseases [21, 28, 38, 39]. Meanwhile, therapeutic strategies targeting UBE2L3 have garnered significant attention in the medical field‌. DMF, a clinically approved drug for multiple sclerosis, has been shown to exert anti-inflammatory effects through UBE2L3, though its precise molecular mechanism remains unclear [29, 38]. Leveraging IVA's electrophilic property, we employed LC–MS/MS to reveal its covalent binding to the Cys86 of UBE2L3. Molecular docking confirmed that the thiol group of UBE2L3's Cys86 forms a stable C-S bond with IVA's C11-C13 unsaturated double bond via Michael addition. Additionally, hydrogen bond between IVA's hydroxyl group and Glu118's carbonyl group, along with hydrophobic interactions between IVA's lactone oxygen and His119's imidazole ring, collectively facilitate IVA's binding to UBE2L3. Dynamics simulations further corroborated these findings, demonstrating that IVA covalently modifies UBE2L3's Cys86 to form a stable complex. Notably, Cys86 is a critical site for UBE2L3's ubiquitin-conjugating enzyme activity [40, 41]. CoIP analysis showed that IVA treatment significantly suppressed LPS-induced K63 and M1 ubiquitination of NEMO. Previous studies have showed that UBE2L3 is a dominant ubiquitin-conjugating enzyme for the K63 and M1 ubiquitination of NEMO, a key event in IKKs complex formation and NF-κB activation [28, 29, 35]. Therefore, we proposed that IVA inhibits NF-κB signaling by covalently modifying UBE2L3's catalytic Cys86, thereby blocking NEMO ubiquitination. While our in vitro data demonstrate that IVA inhibits UBE2L3-mediated NEMO ubiquitination, direct evidence of UBE2L3 enzymatic activity and NEMO ubiquitination in brain tissue following in vivo administration is currently lacking and warrants further investigation. Furthermore, as an E2 enzyme, UBE2L3 requires cooperation with specific E3 ubiquitin ligases to execute its catalytic function. Notably, studies have implicated linear ubiquitin chain assembly complex (LUBAC) in M1-linked ubiquitination of NEMO [38]. Although the specific E3 ligase(s) cooperating with UBE2L3 in this cascade remain to be identified in the context of IVA-mediated inhibition, this represents an interesting direction for future investigation. Previous studies have also highlighted DMF's electrophilic property, enabling it to exert anti-inflammatory and antioxidant effects by modifying target protein cysteine residues [42, 43]. Based on this, we thus speculated that DMF may similarly covalently modify UBE2L3's Cys86, thereby inhibiting NF-κB activation and treating multiple sclerosis. Furthermore, our findings also demonstrated that Cys86 residue serves as a druggable site on UBE2L3, providing a molecular basis for designing UBE2L3 inhibitors. Of note, CETSA and DARTS assays showed that IVA decreased rather than increased the thermal stability and protease resistance of UBE2L3. Given that IVA covalently modifies the catalytic Cys86 of UBE2L3, this interaction may induce conformational perturbations that reduce protein stability and increase susceptibility to proteolytic digestion.

## Limitations

Pharmacokinetic data, including brain penetration and bioavailability, as well as systematic toxicological assessments, are currently lacking; however, in silico prediction using the Deep-B3 blood–brain barrier permeability model [44] suggested that IVA is permeable (Supplementary Fig. 4), though experimental validation is still needed. Notably, covalent targeting has emerged as a promising strategy in drug discovery. Nevertheless, as an electrophilic compound containing an α-methylene-γ-lactone moiety, IVA carries potential off-target risks due to its inherent cysteine reactivity, which warrants careful evaluation in future studies.

## Conclusion

In summary, our study demonstrated that UBE2L3 is the direct functional target of IVA. By covalently binding to Cys86, IVA inhibits UBE2L3-mediated NEMO ubiquitination, thereby effectively suppressing NF-κB activation and neuroinflammation both in vitro and in vivo. Consequently, IVA emerges as a promising lead compound for further preclinical investigation of neuroinflammatory disorders.

## Supplementary Information


Supplementary material 1.

## Data Availability

Data will be made available on reasonable request.

## Abbreviations

AD: Alzheimer’s disease
BME: β-Mercaptoethanol
CETSA: Cellular thermal shift assay
COX-2: Cyclooxygenase 2
CXCL: C-X-C motif chemokine
DARTS: Drug affinity responsive target stability
DMF: Dimethyl fumarate
GEO: Gene expression omnibus
HE: Hematoxylin and eosin
IA: Iodoacetamide
Iba1: Ionized calcium-binding adapter molecule 1
IF: Immunofluorescence
IHC: Immunohistochemistry
IκBα: Inhibitory subunit of NF-κB alpha
IKK: Inhibitor of NF-κB kinase
IL: Interleukin
iNOS: Inducible nitric oxide synthase
IP: Immunoprecipitation
IVA: Ivangustin
JNK: C-Jun N-terminal kinase
K63: Lysine 63
LC–MS: Liquid chromatography-mass spectrum
LPS: Lipopolysaccharide
M1: Methionine 1
MCP-1: Monocyte chemotactic protein-1
NEMO: NF-κB essential modulator
NF-κB: Nuclear factor kappa-B
NO: Nitric oxide
PD: Parkinson’s disease
qRT-PCR: Quantitative real time polymerase chain reaction
TNF-α: Tumor necrosis factor-alpha
Ub: Ubiquitin
UBE2L3: Ubiquitin conjugating enzyme E2L3
