# Supplementary material for: Ivangustin, a sesquiterpene lactone from Inula japonica Thunb., attenuates neuroinflammation through covalent inhibition of ubiquitin-conjugating enzyme UBE2L3
Source: Chin Med. 2026 Sep 2;21:217. doi: 10.1186/s13020-026-01488-9 (PMC13536563; doi:10.1186/s13020-026-01488-9)
Supplement: Supplementary file 1 [file 13020_2026_1488_MOESM1_ESM.docx]

**Supplementary materials**

**Supplementary Figure 1**. **HPLC analysis of ivangustin.** HPLC analysis method: InertSustain C_18_ column; MeCN/H_2_O (0.05%H_3_PO_4_) = 20/80-50/50, 0-35min, 1 mL/min.

**Supplementary Figure 2. Leave-one-out sensitivity analysis for Mendelian randomization of UBE2L3 expression on neurodegenerative disease risk.** (**A**) Parkinson‘s disease (24 SNPs); (**B**) cognitive impairment (22 SNPs); (**C**) Alzheimer’s disease (3 SNPs). The analysis was performed by sequentially removing each SNP and recalculating the causal estimate using the IVW method. The red dashed line indicates the causal estimate using all SNPs; error bars represent 95% confidence intervals. For Parkinson‘s disease and cognitive impairment, all leave-one-out estimates remained consistent with the overall causal estimate, indicating that the results were not driven by any single SNP. For Alzheimer’s disease, the analysis was performed with only 3 SNPs; removal of one SNP shifted the estimate toward the null, reflecting the limited statistical power of this sensitivity analysis. Results for AD should therefore be interpreted with caution.

**Supplementary Figure 3. Total arm entries in the Y-maze test.** Mice were treated with vehicle, IVA (5, 10, or 20 mg/kg), or DMF (100 mg/kg) for four consecutive days, followed by LPS (2.5 mg/kg) injection on day 4. Total arm entries were recorded during the 5-min Y-maze test. Data are presented as mean ± SD. ns, not significant, *p < 0.05, **p < 0.01, ***p < 0.001, **** p < 0.0001, vs. LPS, n = 6.

**Supplementary Figure 4：In silico prediction of blood-brain barrier (BBB) permeability of ivangustin using the Deep-B3 model.** The SMILES string of ivangustin was used as input, and the model predicted it as BBB-permeable. The prediction was performed using the online webserver (<http://cbcb.cdutcm.edu.cn/deepb3/>) based on the Deep-B3 model (Tang et al., 2022).

**Supplementary Table 1**. **PCR primer sequences**

| Gene name | Sequence (5’-3’) |
| --- | --- |
| Mouse iNOS FP | GAG ACA GGG AAG TCT GAA GCA C |
| Mouse iNOS RP | CCA GCA GTA GTT GCT CCT CTT C |
| Mouse COX2 FP | GCG ACA TAC TCA AGC AGG AGC A |
| Mouse COX2 RP | AGT GGT AAC CGC TCA GGT GTT G |
| Mouse IL-1β FP | TGG ACC TTC CAG GAT GAG GAC A |
| Mouse IL-1β RP | GTT CAT CTC GGA GCC TGT AGT G |
| Mouse IL-6 FP | TAC CAC TTC ACA AGT CGG AGG C |
| Mouse IL-6 RP | CTG CAA GTG CAT CAT CGT TGT TC |
| Mouse TNF-α FP | CCA CCA CGC TCT TCT GTC TAC |
| Mouse TNF-α RP | GAG ACA GGG AAG TCT GAA GCA C |
| Mouse MCP-1 FP | TAA AAA CCT GGA TCG GAA CCA AA |
| Mouse MCP-1 RP | GCA TTA GCT TCA GAT TTA CGG GT |
| Mouse CXCL1 FP | ATC CAG AGC TTG AAG GTG TTG |
| Mouse CXCL1 RP | GTC TGT CTT CTT TCT CCG TTA CTT |
| Mouse CXCL9 FP | GGA GTT CGA GGA ACC CTA GTG |
| Mouse CXCL9 RP | GGG ATT TGT AGT GGA TCG TGC |
| Mouse GAPDH FP | CCC ATC ACC ATC TTC CAG GAG C |
| Mouse GAPDH RP | CCA GTG AGC TTC CCG TTC AGC |

**Supplementary Table 2**. **The** **siRNA sequence targeting mouse UBE2L3**

| Gene name | Sequence (5’-3’) |
| --- | --- |
| UBE2L3 siRNA sense | CCU CAU AGC ACU GGU GAA U |
| UBE2L3 siRNA anti-sense | AUU CAC CAG UGC UAU GAG G |
| NC sense | UUC UCC GAA CGU GUC ACG UTT |
| NC anti-sense | ACG UGA CAC GUU CGG AGA ATT |
